# Cost-effectiveness Evaluation of Targeted Surgical and Endoscopic Therapies for Early Colorectal Adenocarcinoma Based on Biomarker Profiles

**DOI:** 10.1001/jamanetworkopen.2019.19963

**Published:** 2020-03-09

**Authors:** Se Ryeong Jang, Han Truong, Aaron Oh, Jin Choi, Angela C. Tramontano, Monika Laszkowska, Chin Hur

**Affiliations:** 1 Department of Epidemiology, Mailman School of Public Health, Columbia University, New York, New York; 2 now with College of Population Health, Thomas Jefferson University, Philadelphia, Pennsylvania; 3 Herbert Irving Comprehensive Cancer Center, Vagelos College of Physicians and Surgeons, Columbia University, New York, New York; 4 Institute for Technology Assessment, Massachusetts General Hospital, Harvard Medical School, Boston; 5 Department of Medicine, New York Presbyterian/Columbia University Medical Center, New York, New York

## Abstract

**Importance:**

Colorectal cancer (CRC) is the second leading cause of cancer-related mortality in the United States. The prognosis for patients with CRC varies widely, but new prognostic biomarkers provide the opportunity to implement a more individualized approach to treatment selection.

**Objective:**

To assess the cost-effectiveness of 3 therapeutic strategies, namely, endoscopic therapy (ET), laparoscopic colectomy (LC), and open colectomy (OC), for patients with T1 CRC with biomarker profiles that prognosticate varying levels of tumor progression in the US payer perspective.

**Design, Setting, and Participants:**

In this economic evaluation study, a Markov model was developed for the cost-effectiveness analysis. Risks of all-cause mortality and recurrent cancer after ET, LC, or OC were estimated with a 35-year time horizon. Quality of life was based on EuroQoL 5 Dimensions scores reported in the published literature. Hospital and treatment costs reflected Medicare reimbursement rates. Deterministic and probabilistic sensitivity analyses were performed. Data from patients with T1 CRC and 6 biomarker profiles that included adenomatous polyposis coli (*APC)*, *TP53* and/or *KRAS*, or *BRAFV600E* were used as inputs for the model. Data analyses were conducted from February 27, 2019, to May 13, 2019.

**Exposures:**

Endoscopic therapy, LC, and OC.

**Main Outcomes and Measures:**

The primary outcomes were unadjusted life-years, quality-adjusted life-years (QALYs), and the incremental cost-effectiveness ratio (ICER) between competing treatment strategies.

**Results:**

Endoscopic therapy had the highest QALYs and the lowest cost and was the dominant treatment strategy for T1 CRC with the following biomarker profiles: *BRAFV600E*, *APC*(1)/*KRAS*/*TP53*, *APC*(2) or *APC*(2)/*KRAS* or *APC*(2)/*TP53*, or *APC*(1) or *APC*(1)/*KRAS* or *APC*(1)/*TP53*. The QALYs gained ranged from 16.97 to 17.22, with costs between $68 902.75 and $77 784.53 in these subgroups. For the 2 more aggressive biomarker profiles with worse prognoses (*APC*(2)/*KRAS*/*TP53* and *APC*wt [wild type]), LC was the most effective strategy (with 16.45 and 16.61 QALYs gained, respectively) but was not cost-effective. Laparoscopic colectomy cost $65 234.87 for *APC*(2)/*KRAS*/*TP53* and $71 250.56 for *APC*wt, resulting in ICERs of $113 290 per QALY and $178 765 per QALY, respectively.

**Conclusions and Relevance:**

This modeling analysis found that ET was the most effective strategy for patients with T1 CRC with less aggressive biomarker profiles. For patients with more aggressive profiles, LC was more effective but was costly, rendering ET the cost-effective option. This study highlights the potential utility of prognostic biomarkers in T1 CRC treatment selection.

## Key Points

**Question:**

What are the cost-effective treatment strategies for patients with T1 colorectal cancer with biomarker profiles that prognosticate varying levels of tumor progression?

**Findings:**

In this economic evaluation study, a Markov model was used to compare the cost-effectiveness of endoscopic therapy, laparoscopic colectomy, and open colectomy for treatment of T1 colorectal cancer stratified by 6 biomarker profiles that included *APC*, *TP53* and/or *KRAS,* or *BRAFV600E.* Laparoscopic colectomy resulted in the greatest quality-adjusted life-years for the 2 more aggressive biomarker profiles but was expensive; for all patients, endoscopic therapy was the cost-effective option.

**Meaning:**

Tailoring colorectal cancer treatment to a patient’s biomarker profile may result in both better health outcomes and health care cost savings.

## Teaser

This economic evaluation study assesses the cost-effectiveness of 3 therapeutic strategies (endoscopic therapy, laparoscopic colectomy, and open colectomy) for patients with T1 colorectal cancer with biomarker profiles that predict varying levels of tumor progression in the US payer perspective.

## Introduction

Colorectal cancer (CRC) is the fourth most prevalent cancer and the second leading cause of cancer-related mortality in the United States.^1^ Despite declines in recent decades, approximately 140 000 individuals in the United States are diagnosed as having CRC every year, with one-third of them surviving less than 5 years.^2^ The current CRC staging system is mostly based on the anatomic features of the tumor, such as intestinal wall and peritoneal infiltration (T1-T4), the number of involved regional lymph nodes (N0-N2), and occurrence of distant metastasis (M), which are insufficient for accurate prognostic prediction or clinical management.^3,4^ Recent advances in genomic profiling of this molecularly complex disease and in treatment of T1 CRC lesions could help develop more personalized treatment strategies and further lower the CRC mortality rate.

Although CRC is a heterogeneous disease that is associated with multiple genetic factors, its tumor initiation and progression depend on mutations in a few key drivers, such as adenomatous polyposis coli (*APC* [OMIM 611731], found in 85% of CRC tumors), *TP53* (OMIM 191170) (found in 35%-55% of tumors), *KRAS* (OMIM 190070) (found in 35%-45% of tumors), and *BRAFV600E* (OMIM 164757) (found in 8%-12% of tumors).^5,6^ Inactivation of *APC* initiates the adenoma-carcinoma pathway for microsatellite-stable tumors, with these adenomas advancing to CRC when additional mutations are present in *TP53*, *KRAS*, or both.^6,7,8,9^ In contrast, wild-type *APC* (*APC*wt) and *BRAFV600E* mutations are associated with microsatellite instability–high (MSI-H) tumors.^7^

A 2016 study by Schell et al^7^ demonstrated that overall CRC survival varies substantially based on the number of truncating mutations in *APC*. For example, tumors with 1 mutation in *APC*, 1 mutation in *APC* partnering with a *KRAS* mutant, or 1 mutation in *APC* partnering with a *TP53* mutant (hazard ratio [HR], 1.00) have better outcomes than tumors with 2 mutations in *APC*, 2 mutations in *APC* and a *KRAS* mutant, or 2 mutations in *APC* and a *TP53* mutant (HR, 1.11). Tumors with 1 mutation in *APC* coexisting with *KRAS* and *TP53* mutants have worse survival (HR, 1.48), followed by tumors with *APC*wt (HR, 1.94) and, ultimately, tumors with 2 mutations in *APC* partnering with *KRAS* and *TP53* mutants (HR, 2.48). It is possible that optimal treatment strategies differ between these subgroups, although no studies, to our knowledge, have investigated this question. Treatments that take the prognosis of each biomarker profile into consideration could optimize clinical outcomes and resource use.

The current national guidelines for treatment of nonmetastatic T1 CRC tumors or high-risk polyps recommend colectomy.^10^ Yet, studies^11,12,13^ demonstrate that most colon neoplasms could also be safely and effectively removed with endoscopic resection techniques, such as endoscopic mucosal resection and endoscopic submucosal dissection. Given that endoscopic resections cost substantially less than the average cost of a colectomy, identifying patients who would most benefit from an endoscopic approach to resection would be crucial in reducing health care costs.

Model-based analyses can provide a framework for estimating long-term clinical benefits and cost-effectiveness associated with various resection procedures in patients with different tumor mutation profiles. Therefore, a decision analytic Markov model was used to compare unadjusted life-years, quality-adjusted life-years (QALYs), and cost-effectiveness of endoscopic therapy (ET) vs laparoscopic colectomy (LC) vs open colectomy (OC) for patients with early or T1 CRC with various biomarker mutation profiles at varying ages. The ultimate objective for this study was to identify the optimal therapy for different classes of patients with T1 CRC.

## Methods

### Model Overview

In this economic evaluation study, we developed a Markov model using TreeAge Pro 2019 (TreeAge Software, Inc) to assess the cost-effectiveness of ET, LC, and OC. Given that the median age at CRC diagnosis is 67 years,^14^ we chose to model 65-year-old patients with T1N0M0, T1N1M0, and T1N2M0 CRC (ie, histologic tumor invasion through the muscularis mucosa and into, but not beyond, the submucosa and amenable to surgical cure^15^) not previously treated with radiation therapy or chemotherapy as the base case population. The base case population was classified into 6 different biomarker profiles (Table 1). Because this was an in silico mathematical model that involved only the analysis of deidentified data within a publicly available data set, it did not require the institutional review board’s review or individual informed consent as per guidelines of the institutional review board at Columbia University Irving Medical Center. This evaluation followed all criteria of the Consolidated Health Economic Evaluation Reporting Standards (CHEERS) reporting guidelines.

**Table 1.** Biomarker Profiles, With Respective Mortality Rates

Class	Biomarker	Description	Mortality Rate, No. of Deaths /10 000 Individuals per mo^a^
0	*APC*wt	Wild-type *APC*	74
1	*APC*(1)	1 Mutation in *APC*	12
	*APC*(1)/*KRAS*	1 Mutation in *APC* partnering with a *KRAS* mutant	
	*APC*(1)/*TP53*	1 Mutation in *APC* partnering with a *TP53* mutant	
2	*APC*(2)	2 Mutations in *APC*	24
	*APC*(2)/*KRAS*	2 Mutations in *APC* partnering with a *KRAS* mutant	
	*APC*(2)/*TP53*	2 Mutations in *APC* partnering with a *TP53* mutant	
3	*APC*(1)/*KRAS*/*TP53*	1 Mutation in *APC* partnering with *KRAS* and *TP53* mutants	25
4	*APC*(2)/*KRAS*/*TP53*	2 Mutations in *APC* partnering with *KRAS* and *TP53* mutants	119
MSI-H	*BRAFV600E* mutant	Not applicable	28

Abbreviations: MSI-H, microsatellite instability–high; wt, wild type.

^a^ The mortality rates are specific to colorectal cancer. These values were calibrated using data reported by Schell et al^7^ and Malesci et al.^17^

**Figure 1.**
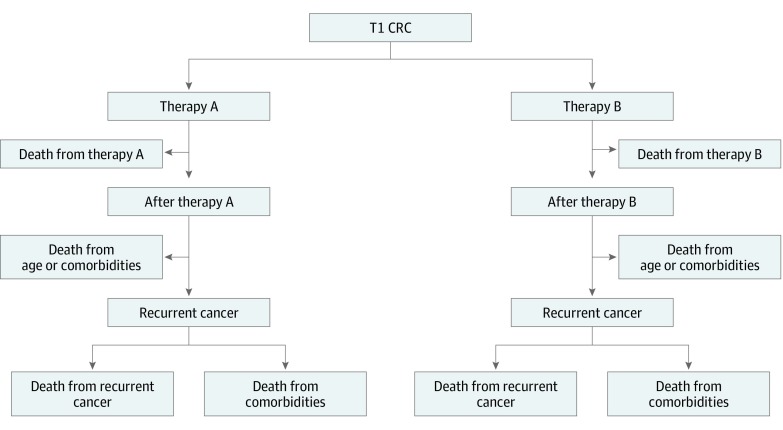
Simplified Model Schematic Strategy 1 is therapy A (endoscopic therapy) and therapy B (emergency colectomy). Strategy 2 is therapy A (laparoscopic colectomy) and therapy B (emergency open colectomy). Strategy 3 is therapy A (open colectomy) and no therapy B. CRC indicates colorectal cancer.

Hypothetical patient cohorts could undergo ET, LC, or OC in the simulation model. If the procedure was successful, patients would enter a postprocedural state in which they underwent surveillance colonoscopy 1 year after the procedure and subsequently every 3 years.^10^ If complications arose during ET or LC, patients would undergo emergency OC and either die or enter the postemergency colectomy state. Regardless of the type of treatment, all patients were at risk of recurrent cancer and could die of either complications of recurrence or age-related comorbidities (Figure 1).

The model’s cycle length was 1 month, and patients continued in the model from age 65 years until death or age 100 years, whichever came first (35-year time horizon). All-cause mortality was based on the 2015 US life tables from the Centers for Disease Control and Prevention.^16^

### Biomarker Profiles

Class 0 represented patients with *APC*wt. Class 1 represented patients with 1 truncating mutation in *APC* (*APC*(1)), 1 mutation in *APC* partnering with a *KRAS* mutant (*APC*(1)/*KRAS*), or 1 mutation in *APC* partnering with a *TP53* mutant (*APC*(1)/*TP53*). Class 2 represented patients with 2 mutations in *APC* (*APC*(2)), 2 mutations in *APC* partnering with a *KRAS* mutant (*APC*(2)/*KRAS*), or 2 mutations in *APC* partnering with a *TP53* mutant (*APC*(2)/*TP53*). Class 3 represented patients with 1 mutation in *APC* partnering with *KRAS* and *TP53* mutants (*APC*(1)/*KRAS*/*TP53*). Class 4 represented patients with 2 mutations in *APC* partnering with *KRAS* and *TP53* mutants (*APC*(2)/*KRAS*/*TP53*). The MSI-H class represented patients with mutations in *BRAFV600E* (Table 1).

### Parameter Estimates

Data from the published literature on patients with T1 CRC at the mean age of 65 years were used as inputs for the model.^7,17,18,19,20,21,22,23,24,25,26,27,28^ Hospital and treatment costs reflected Medicare reimbursement rates. Quality of life was based on EuroQol 5 Dimensions^29,30,31,32^ scores reported in the published literature.^33,34,35,36,37^ Table 2 lists base-case values and ranges used in sensitivity analyses.^7,15,16,17,18,19,20,21,22,23,24,25,26,27,28,29,30,31,32,33,34,35,36,37^

**Table 2.** Base-Case Inputs and Ranges Used in Sensitivity Analyses^a^

Parameter	Base-Case Estimate (Range)	Distribution	Source
Age, y	65 (55-75)	Uniform	CDC US life tables,^16^ 2015
Sex	Female, male	Female, male	
**Probability**			
30-d mortality after procedure^b^			
ET	0.0001 (0.000075-0.000125)	β	Pox et al,^19^ 2012
			Ahlenstiel et al,^20^ 2014
OC, emergency OC	0.0100 (0.0075-0.0125)	β	Nelson et al,^21^ 2004
LC	0.0050 (0.00375-0.00625)	β	Nelson et al,^21^ 2004
Complications			
ET	0.0100 (0.0075-0.0125)	β	Tanaka et al,^22^ 2007
OC, emergency OC	0.0500 (0.0375-0.0625)	β	COLOR Study Group,^18^ 2005
LC	0.0400 (0.03-0.05)	β	COLOR Study Group,^18^ 2005
Conversion			
From ET to OC	0.0150 (0.0113-0.0188)	β	Paramasivam et al,^23^ 2013
			Draganov,^24^ 2018
From LC to OC	0.1900 (0.1425-0.2375)	β	COLOR Study Group,^18^ 2005
Recurrent cancer			
ET	0.0012 (0.0009-0.0015)	β	Fujihara et al,^25^ 2017
			Yoda et al,^26^ 2013
OC, emergency OC	0.0006 (0.00045-0.00075)	β	Fujihara et al,^25^ 2017
			Asayama et al,^27^ 2016
LC	0.0007 (0.000525-0.000875)	β	Fujihara et al,^25^ 2017
			Fleshman et al,^28^ 2007
Mortality^b^			
Class 4	0.0119 (0.0089-0.0149)	β	Schell et al,^7^ 2016 and Malesci et al,^17^ 2007
Class 0	0.0074 (0.0056-0.0093)	β	
MSI-H	0.0028	NA	
Class 3	0.0025	NA	
Class 2	0.0024	NA	
Class 1	0.0012	NA	
**Cost, $**			
Procedure			
ET	980 (490-1960)	γ	Boston Scientific,^30^2019
OC	44 837 (22 419-89 674)	γ	AHRQ^29^
			Sheetz et al,^31^ 2017
LC	37 778 (18 889-75 556)	γ	AHRQ^29^
			Sheetz et al,^31^ 2017
Emergency OC from ET	43 857 (21 929-87 714)	γ	NA
Emergency OC from LC	7059 (3530-14 118)	γ	NA
Surveillance colonoscopy	28 (14-56) per mo	γ	Boston Scientific,^30^2019
Recurrent cancer treatment	3872 (1936-7744) per mo	γ	Saini et al,^32^ 2010
Complications			
Delayed bleeding	7493 (3747-14 986)	γ	AHRQ^29^ (*ICD-10* K92.2)
Bowel obstruction >3 d	5671 (2836-11 342)	γ	AHRQ^29^ (*ICD-10* K56.60)
**Utility**			
Before procedure	0.88 (0.792-0.968)	Uniform	Dang et al,^33^ 2019
			Chen et al,^35^ 2015
			Jeong and Cairns,^36^ 2016
After ET, 0-1 mo	0.82 (0.738-0.902)	β	Kapidzic et al,^37^ 2012
After ET, >1 mo	0.95 (0.855-1.000)	β	NA
After OC, 0-1 mo	0.79 (0.711-0.869)	Uniform	Wilson et al,^34^ 2006
After OC, >1 mo	0.91 (0.819-1.000)	β	NA
After LC, 0-1 mo	0.81 (0.729-0.891)	β	NA
After LC, >1 mo	0.93 (0.837-1.000)	β	Dang et al,^33^ 2019
After emergency OC, 0-1 mo	0.74 (0.666-0.814)	β	Wilson et al,^34^ 2006
After emergency OC, >1 mo	0.91 (0.819-1.000)	β	NA
Recurrent cancer	0.75 (0.675-0.825)	β	Jeong and Cairns,^36^ 2016

Abbreviations: AHRQ, Agency for Healthcare Research and Quality; CDC, Centers for Disease Control and Prevention; COLOR, Colon Cancer Laparoscopic or Open Resection; ET, endoscopic therapy; *ICD-10*, *International Statistical Classification of Diseases and Related Problems*, *Tenth Revision*; LC, laparoscopic colectomy; MSI-H, microsatellite instability–high; NA, not applicable; OC, open colectomy.

^a^ Refer to the Methods subsection Model Transition Probabilities and Calibration for an explanation on how mortality rates were calibrated.

^b^ Mortality rates are specific to colorectal cancer. The classes are listed in order from worst prognosis to best prognosis.

### Model Transition Probabilities and Calibration

Cancer-specific survival for T1 CRC with the biomarker profiles and treatment strategies of interest has not been reported, to our knowledge. Therefore, estimates of cancer-specific deaths for the 6 different biomarker profiles were calculated based on data published by Schell et al^7^ in 2016 and by Malesci et al^17^ in 2007. Given that Schell et al^7^ reported all stages of CRC rather than T1 CRC alone, we used the HRs reported for patients with CRC at each level of tumor invasion (T1-T4) by Malesci et al^17^ (HR, 1.00 [stage 1], 2.11 [stage 2], 7.99 [stage 3], and 52.2 [stage 4]) to adjust HRs for each of the biomarker profiles reported by Schell et al^7^ (HR, 1.94 [class 0], 1.00 [class 1], 1.11 [class 2], 1.48 [class 3], and 2.48 [class 4]). To ensure that our estimates represent the cancer-specific mortality rate for patients with T1 CRC who have undergone endoscopy or colectomy, we compared our calibrated values with mortality rates generated from the Surveillance, Epidemiology, and End Results–Medicare database^38^ for external validation (eFigure 1 in the Supplement). We also generated Kaplan-Meier curves for each biomarker profile for patients with CRC at all cancer stages by weighting the estimated biomarker profile–specific mortality with the proportion of each stage of CRC represented in the study population by Schell et al.^7^ These Kaplan-Meier curves were calibrated to those published by Schell et al^7^ (eFigure 2 in the Supplement). The cancer-specific mortality rates predicted for the 6 biomarker profiles for T1 CRC are listed in Table 1.

The postprocedural survival and recurrent cancer rates for ET in our model were represented by the mean of those for polypectomy, endoscopic mucosal resection, and endoscopic submucosal dissection. For the complication rate for ET, we used the mean complication rate for endoscopic submucosal dissection. Complications were sequelae requiring readmission (delayed bleeding for ET and delayed bleeding and >3 days of bowel obstruction for LC and OC) for all treatment strategies.^29^

We assumed that the recurrent cancer rate and quality of life for those who underwent an emergency OC were the same as for those who underwent an elective OC. All patients who underwent the same treatment were assigned an equal chance of developing recurrent cancer, with the assumption that this possibility was true regardless of their biomarker profiles.

### Costs and Quality-of-Life Adjustments

We assumed the US payer perspective. Costs were based on the published literature^30,31,32^ and the Agency for Healthcare Research and Quality’s Healthcare Cost and Utilization Project database.^29^ All values were converted to 2019 US dollars using the Consumer Price Index^39^ to account for inflation and the differences in the time when the costs were reported. We used Medicare national mean payment rates for all treatment strategies. For LCs and OCs, we included total episode payments, index hospitalization payments, and physician payments to account for the postprocedural inpatient care.^30,31^ The net difference between ET and OC procedural costs was used for the cost of emergency colectomy after ET. The same method was applied for calculating the cost of emergency OC after LC. To account for the greater uncertainty regarding the use of health care resources, costs were varied through a total range covering half the base-case estimate cost at the low end and twice the base-case estimate cost at the high end in the 1-way sensitivity analyses.

Quality-of-life measures were adjusted to utility scores for the specific health states. Although a utility score of 0.88 was assigned to all patients before their procedure, different disutility values were applied for each treatment group for the first month after the procedure to reflect the number of productive days the patients would lose from being hospitalized (0 days for ET, 8 days for LC, and 9 days for OC).^18,33,34^ We counted 1 day spent at the hospital as 30% of a full productive day lived. Both costs and utilities were discounted at an annual rate of 3%.^40^

### Outcomes

The primary outcomes were unadjusted life-years, QALYs, and the incremental cost-effectiveness ratio (ICER) among competing treatment strategies. The willingness-to-pay (WTP) threshold was set at $100 000 per QALY.^41^

### Statistical Analysis

A base-case analysis using best estimates for all model parameters was performed separately for T1 CRC with the 6 biomarker profiles. One-way sensitivity analyses and deterministic and probabilistic sensitivity analyses were performed for the 2 biomarker profiles (classes 0 and 4), for which LC was the most effective strategy but not the cost-effective one. One-way sensitivity analyses were conducted to assess changes in which variables would render LC cost-effective at the threshold of $100 000 per QALY for classes 0 and 4. Data analyses were conducted from February 27, 2019, to May 13, 2019.

## Results

### Base-Case Analysis

Endoscopic therapy had the highest QALYs and lowest cost and was the dominant treatment strategy for T1 CRC with the following biomarker profiles: MSI-H and classes 1, 2, and 3. The results of the base-case analysis are listed in Table 3, where the biomarker profiles are listed in the order of highest to lowest mortality rate. For the 2 more aggressive biomarker profiles with worse prognoses (*APC*(2)/*KRAS*/*TP53* and *APC*wt) (ie, classes 0 and 4), unadjusted life-years gained were highest for OC among the 3 treatment strategies tested (18.08 for class 0 and 17.89 for class 4); however, the highest QALYs gained were for LC (16.61 for class 0 and 16.45 for class 4). For the less aggressive biomarker profiles (ie, classes 1, 2, and 3 and MSI-H), unadjusted life-years gained were highest for LC (18.63, 18.48, 18.47, and 18.44, respectively); however, the highest QALYs gained were for ET (17.22, 17.03, 17.01, and 16.97, respectively). Costs ranged between $68 902.75 and $77 784.53 in these 4 subgroups.

**Table 3.** Base-Case Results for Patients With T1 Colorectal Cancer, by Biomarker Profile^a^

Class/Therapy	Unadjusted Life-Years	Change in Unadjusted Life-Years^**b**^	Cost, $	QALYs
**Class 4**				
ET	17.31	NA	41 444	16.24
LC^c^	17.86	0.55	65 235	16.45
OC	17.89	0.03	68 619	16.22
**Class 0**				
ET	17.66	NA	51 586	16.50
LC^d^	18.07	0.41	71 251	16.61
OC	18.08	0.01	73 934	16.36
**MSI-H**				
ET	18.29	NA	68 903	16.97
LC	18.44	0.15	81 494	16.89
OC	18.41	−0.03	82 980	16.61
**Class 3**				
ET	18.34	NA	70 423	17.01
LC	18.47	0.13	82 391	16.91
OC	18.44	−0.03	83 773	16.63
**Class 2**				
ET	18.36	NA	70 943	17.03
LC	18.48	0.12	82 699	16.92
OC	18.45	−0.03	84 044	16.64
**Class 1**				
ET	18.62	NA	77 785	17.22
LC	18.63	0.01	86 737	17.03
OC	18.58	−0.05	87 609	16.74

Abbreviations: ET, endoscopic therapy; LC, laparoscopic colectomy; MSI-H, microsatellite instability–high; NA, not applicable; OC, open colectomy; QALY, quality-adjusted life-year.

^a^ Costs and QALYs were discounted at 3% per year.

^b^ The calculation is based on all-cause mortality.

^c^ The change in cost was $23 790, and the change in QALY was 0.21.

^d^ The change in cost was $19 664, and the change in QALY was 0.11.

Among all of the biomarker profiles, cost was lowest for the ET strategy and highest for the OC strategy. Given that ET was the cheapest treatment option that yielded the most QALYs gained for classes 1, 2, and 3 and MSI-H, ET appears to be the clinically superior and cost-saving treatment strategy for these profiles.

For classes 0 and 4, ET and LC were competing strategies considering that the most QALYs were gained from LC, whereas the lowest cost was incurred from ET. For class 4, the ICER for comparing ET with LC was $113 290 per QALY for class 4. Therefore, LC was not cost-effective at the threshold of $100 000 per QALY. Similarly, for class 0, the ICER of $178 765 per QALY for *APC*wt was not cost-effective. Consequently, ET emerged as the cost-effective option for both classes 0 and 4.

### Sensitivity Analyses

**Figure 2.**
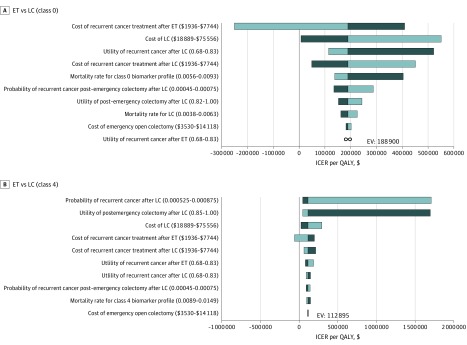
Ten Most Important Variables for Each of the Biomarker Profiles A and B, Figures demonstrate the univariate change on the incremental cost-effectiveness ratio (ICER) of laparoscopic colectomy (LC) vs endoscopic therapy (ET) for classes 0 and 4 in the order of decreasing sensitivity. The dividing line between the two different shades of blue bars denotes the base-case ICER. The darker blue bar represents the ICER range when the parameter is lower than its base-case value. The lighter blue bar represents the ICER range when the parameter is higher than its base-case value. ∞ indicates ICER when values for utility of recurrent cancer after endoscopic therapy (ET) are varied; EV, expected value (baseline); ICER, incremental cost-effectiveness ratio; LC, laparoscopic colectomy; and QALY, quality-adjusted life-year.

The 10 most important variables for each of the biomarker profiles are shown in tornado diagrams (Figure 2). For class 0, the results were most sensitive to the cost of recurrent cancer treatment after ET or after LC, utility of recurrent cancer after ET, cost of LC, and biomarker-specific probability of death. For class 4, the preferred strategy changed based on the probability of recurrent cancer after LC and the utility of emergency colectomy after LC.

At the WTP threshold of $100 000, ET would be the cost-effective strategy over LC for class 0 if the cost of recurrent cancer treatment after ET was less than $4600 per month (eFigure 3 in the Supplement). However, if the cost of recurrent cancer treatment after LC was less than $2600 per month, then LC would be the preferred strategy (eFigure 4 in the Supplement). Also, LC could be the preferred strategy if the treatment costs were less than $28 000 (eFigure 5 in the Supplement).

For class 4, LC would be the cost-effective strategy over ET if the monthly recurrent cancer rate for those who underwent LC was less than 0.064% or if the rate of those who underwent emergency colectomy during LC was less than 0.051% (eFigure 6 and eFigure 7 in the Supplement). In addition, LC could be cost-effective if the cost of LC was less than $35 000 (eFigure 8 in the Supplement). Furthermore, LC became the dominant strategy over ET when recurrent cancer treatment costs after LC were less than $3400 per month or when costs of recurrent cancer treatment after ET were greater than $4200 per month (eFigure 9 and eFigure 10 in the Supplement). If utility for those who underwent ET but subsequently developed recurrent cancer was less than 0.73 or if utility for those who underwent LC but subsequently developed recurrent cancer was greater than 0.79, then LC would be cost-effective (eFigure 11 and eFigure 12 in the Supplement). In contrast, ET would be the preferred strategy over LC if the utility for emergency colectomy during LC was less than 0.93 or if the monthly mortality rate for class 4 was less than 0.146% (eFigure 13 and eFigure 14 in the Supplement).

The probabilistic sensitivity analyses results were plotted to show the incremental cost-effectiveness of 10 000 trials from a Monte Carlo simulation for classes 0 and 4 (eFigure 15 and eFigure 17 in the Supplement). At the WTP threshold of $50 000, LC was the cost-effective strategy 32% of the time, whereas ET was the cost-effective strategy 38% of the time at the WTP threshold of $50 000 for class 0 (eFigure 16 in the Supplement). For class 4, LC was the cost-effective strategy approximately 34% of the time, whereas ET was the cost-effective strategy 36% of the time for class 4 (eFigure 18 in the Supplement). At and beyond the WTP threshold of $60 000, LC was more likely to be the cost-effective strategy than ET for both biomarker profiles.

## Discussion

For T1 CRC with biomarker profiles in classes 1, 2, and 3 and MSI-H, our model suggests that ET is the cost-effective treatment strategy. Although LC yielded slightly more unadjusted life-years than ET for these biomarker profiles, QALYs were greater in the ET group for all of these biomarker profiles and would be the more comprehensive outcome because it incorporates quality of life associated with treatment in addition to life-years gained.

The modeling analysis also suggested that for T1 CRC with biomarker profiles in class 0 or class 4, LC was the most effective strategy but was not cost-effective. These results were sensitive to recurrent cancer rates, LC treatment cost, quality of life for patients with recurrent cancer, quality of life after emergency colectomy, and mortality rates for specific biomarker profiles. The results underscore the need for long-term outcomes research in patients with T1 CRC with specific biomarker profiles to be able to more accurately predict optimal treatment strategies.

### Strengths and Limitations

This study had several strengths. To our knowledge, this is the first study to use a Markov model to assess the cost-effectiveness of various treatment strategies in T1 CRCs with different tumor biomarker profiles. Another strength of our analysis stems from the ability to risk-stratify T1 CRC and estimate mortality rates for individual tumor biomarker profiles based on published research.^7^ Although multiple studies^5,6,7,8,9,17^ have reported that *APC*, *TP53*, *KRAS*, and *BRAFV600E* were key drivers of CRC, the study by Schell et al^7^ was the first to demonstrate multigene interaction between these genetic factors and their effect on overall survival. Our study built on those findings to identify a targeted, cost-effective treatment strategy for this molecularly complex disease based on specific prognostic biomarkers.

Our results suggest the possibility that providing a biomarker-based treatment approach for patients with T1 CRC could improve outcomes and drive down health care costs; however, further prospective studies are needed to validate these results in the clinical setting. Future studies should investigate potential applications of such a targeted approach to other areas of cancer care, such as personalization of screening and surveillance algorithms by genetic estimates of cancer risk.

We also acknowledge several limitations of our modeling analysis. As with all simulation models, we had to make several assumptions in model design. Although recurrence rates may differ depending on biomarker profile, we did not have specific data to model these nuances. Instead, by assuming that recurrence rates varied by the type of therapy, we could incorporate treatment-specific safety and effectiveness data into the model.

The model did not account for potential extended treatment for patients with poor response to initial therapy. Our patient population of focus was diagnosed at early stages of CRC, and current treatment strategies are highly effective. Although we suspect that this drawback would have minimal effects on outcomes, it is possible that additional therapy may add to the costs of treatment and possibly improve effectiveness. Nonetheless, this factor was beyond the scope of the present analysis.

Other indirect costs not included in our model may have a role in treatment decisions and could alter outcomes. For example, our model excluded the costs of adjunctive therapies, such as chemotherapy, radiation therapy, or palliative care, which are more likely to be used in patients who do not undergo colectomy. It is possible that these costs could bias the model in favor of ET. We also excluded the costs incurred from lost productive days in the initial weeks after treatment, which may increase the ICER between ET and LC.

Although the overall survival for the 6 biomarker profiles analyzed in this study was based on published data reported by Schell et al,^7^ we acknowledge that additional studies are needed to confirm these mortality differences. These data represented overall survival for all stages of CRC for each biomarker profile that received any treatment approach; as a result, we had to calibrate these values to ascertain survival limited to T1 CRC after ET, LC, or OC using HRs reported by Malesci et al.^17^ We validated our calibrated values with the cancer-specific mortality rates for T1 CRC treated with ET and colectomy in the Surveillance, Epidemiology, and End Results registry, which provided reassurance regarding accurate calibration of these data for the model. Nonetheless, our estimates represent an extrapolation of data initially reported for a broader population, and the model could be strengthened by studies that provide additional data on outcomes of these biomarker profiles specifically for T1 CRC.

Although data for LC and OC were based on randomized clinical trials, data for ET were mostly based on retrospective observational studies largely because of the recent advent of ET as a treatment option for T1 CRC. Consequently, the reported estimates for outcomes of ET varied more than those for LC and OC. Because of these parameter uncertainties, we carefully explored the implications of different outcome estimates (eg, posttreatment complication and mortality rates) for the cost-effectiveness outcomes via sensitivity analyses and found that these estimates of ET outcomes did not appear to substantially alter the conclusions of our analyses.

In addition, recurrent cancer rates for LC and OC were extrapolated from a single study^27^ that only reported combined outcomes for both procedures. Our sensitivity analyses demonstrated that the rate of cancer recurrence is an influential factor when comparing optimal treatment outcomes in the model. Therefore, further research in this area will be crucial to better understand these treatment outcomes.

Despite these limitations, data from the Social Security Administration (SSA)^42^ provide external validation for the results of our analysis. According to the SSA, a man entering the SSA system at age 65 years today can expect to live until age 84 years, and his female counterpart can expect to live until age 86.5 years. Therefore, the mean life expectancy for an adult in the United States is 85.3 years. Our base-case population is 65 years old, and unadjusted life-years gained across the 6 biomarker profiles range from 17.31 to 18.63 (Table 3), demonstrating that the 3 treatments examined afford patients near-normal life expectancies of ages 82.31 to 83.63 years. Our findings align with those reported by Soerjomataram et al,^43^ which illustrated that survivors of stage 1 CRC could have a normal life expectancy.

## Conclusions

The findings of the present study suggest that ET is the cost-effective treatment strategy for T1 CRC with biomarker profiles in classes 1, 2, 3, or MSI-H. In contrast, for T1 CRC with biomarker profiles in class 0 or 4, LC may be the more effective strategy, but it is not cost-effective. Choice of therapy in class 0 or 4, which have a worse prognosis, may be dependent on patients’ long-term health outcomes, such as recurrent cancer rates and mortality rates, thus requiring further study. Future prospective studies with adequate patient numbers and follow-up duration are needed to better define and validate targeted treatment approaches in T1 CRC based on tumor biomarker profiles.

## Notes

Supplement. **eFigure 1.** SEER’s CRC T1 Survival Curves With Linear Fit as Mortality Rates **eFigure 2.** Calibrated Survival Curves Over Survival Curves From Schell et al **eFigure 3.** One-Way Sensitivity Analysis Between Recurrent Cancer Treatment Cost Post-ET vs ICER (Class 0) **eFigure 4.** One-Way Sensitivity Analysis Between Cost for Recurrent Cancer Treatment Post-LC vs ICER (Class 0) **eFigure 5.** One-Way Sensitivity Analysis Between LC Treatment Cost vs ICER (Class 0) **eFigure 6.** One-Way Sensitivity Analysis Between Monthly Recurrent Cancer Rate Post-LC vs ICER (Class 4) **eFigure 7.** One-Way Sensitivity Analysis Between Monthly Recurrent Cancer Rate Post–Emergency Colectomy From LC vs ICER (Class 4) **eFigure 8.** One-Way Sensitivity Analysis Between LC Cost vs ICER (Class 4) **eFigure 9.** One-Way Sensitivity Analysis Between Cost for Recurrent Cancer Treatment Post-LC vs ICER (Class 4) **eFigure 10.** One-Way Sensitivity Analysis Between Cost for Recurrent Cancer Treatment Post-ET vs ICER (Class 4) **eFigure 11.** One-Way Sensitivity Analysis Between Cost for Utility for Recurrent Cancer Post-ET vs ICER (Class 4) **eFigure 12.** One-Way Sensitivity Analysis Between Cost for Utility for Recurrent Cancer Post-LC vs ICER (Class 4) **eFigure 13.** One-Way Sensitivity Analysis Between Cost for Utility for Post–Emergency Colectomy From LC vs ICER (Class 4) **eFigure 14.** One-Way Sensitivity Analysis Between Monthly Mortality Rate vs ICER (Class 4) **eFigure 15.** Probabilistic Sensitivity Analysis–ICER Scatterplot (Class 0) **eFigure 16.** Probabilistic Sensitivity Analysis–Cost-effectiveness Acceptability Curve (Class 0) **eFigure 17.** Probabilistic Sensitivity Analysis–ICER Scatterplot (Class 4) **eFigure 18.** Probabilistic Sensitivity Analysis–Cost-effectiveness Acceptability Curve (Class 4)
