# Supplementary material for: Cost-effectiveness Evaluation of Targeted Surgical and Endoscopic Therapies for Early Colorectal Adenocarcinoma Based on Biomarker Profiles
Source: JAMA Netw Open. 2020 Mar 9;3(3):e1919963. doi: 10.1001/jamanetworkopen.2019.19963 (PMC7063501; doi:10.1001/jamanetworkopen.2019.19963)
Supplement: Supplement. — eFigure 1. SEER’s CRC T1 Survival Curves With Linear Fit as Mortality Rates eFigure 2. Calibrated Survival Curves Over Survival Curves From Schell et al eFigure 3. One-Way Sensitivity Analysis Between Recurrent Cancer Treatment Cost Post-ET vs ICER (Class 0) eFigure 4. One-Way Sensitivity Analysis Between Cost for Recurrent Cancer Treatment Post-LC vs ICER (Class 0) eFigure 5. One-Way Sensitivity Analysis Between LC Treatment Cost vs ICER (Class 0) eFigure 6. One-Way Sensitivity Analysis Between Monthly Recurrent Cancer Rate Post-LC vs ICER (Class 4) eFigure 7. One-Way Sensitivity Analysis Between Monthly Recurrent Cancer Rate Post–Emergency Colectomy From LC vs ICER (Class 4) eFigure 8. One-Way Sensitivity Analysis Between LC Cost vs ICER (Class 4) eFigure 9. One-Way Sensitivity Analysis Between Cost for Recurrent Cancer Treatment Post-LC vs ICER (Class 4) eFigure 10. One-Way Sensitivity Analysis Between Cost for Recurrent Cancer Treatment Post-ET vs ICER (Class 4) eFigure 11. One-Way Sensitivity Analysis Between Cost for Utility for Recurrent Cancer Post-ET vs ICER (Class 4) eFigure 12. One-Way Sensitivity Analysis Between Cost for Utility for Recurrent Cancer Post-LC vs ICER (Class 4) eFigure 13. One-Way Sensitivity Analysis Between Cost for Utility for Post–Emergency Colectomy From LC vs ICER (Class 4) eFigure 14. One-Way Sensitivity Analysis Between Monthly Mortality Rate vs ICER (Class 4) eFigure 15. Probabilistic Sensitivity Analysis–ICER Scatterplot (Class 0) eFigure 16. Probabilistic Sensitivity Analysis–Cost-effectiveness Acceptability Curve (Class 0) eFigure 17. Probabilistic Sensitivity Analysis–ICER Scatterplot (Class 4) eFigure 18. Probabilistic Sensitivity Analysis–Cost-effectiveness Acceptability Curve (Class 4) [file jamanetwopen-3-e1919963-s001.pdf]

Supplementary Online Content

Jang SR, Truong H, Oh A, et al. Cost-effectiveness evaluation of targeted surgical and endoscopic therapies for early colorectal adenocarcinoma based on biomarker profiles. *JAMA Netw Open*. 2020;3(3):e1919963. doi:10.1001/jamanetworkopen.2019.19963

eFigure 1. SEER's CRC T1 Survival Curves With Linear Fit as Mortality Rates
eFigure 2. Calibrated Survival Curves Over Survival Curves From Schell et al
eFigure 3. One-Way Sensitivity Analysis Between Recurrent Cancer Treatment Cost Post-ET vs ICER (Class 0)
eFigure 4. One-Way Sensitivity Analysis Between Cost for Recurrent Cancer Treatment Post-LC vs ICER (Class 0)
eFigure 5. One-Way Sensitivity Analysis Between LC Treatment Cost vs ICER (Class 0)
eFigure 6. One-Way Sensitivity Analysis Between Monthly Recurrent Cancer Rate Post-LC vs ICER (Class 4)
eFigure 7. One-Way Sensitivity Analysis Between Monthly Recurrent Cancer Rate Post–Emergency Colectomy From LC vs ICER (Class 4)
eFigure 8. One-Way Sensitivity Analysis Between LC Cost vs ICER (Class 4)
eFigure 9. One-Way Sensitivity Analysis Between Cost for Recurrent Cancer Treatment Post-LC vs ICER (Class 4)
eFigure 10. One-Way Sensitivity Analysis Between Cost for Recurrent Cancer Treatment Post-ET vs ICER (Class 4)
eFigure 11. One-Way Sensitivity Analysis Between Cost for Utility for Recurrent Cancer Post-ET vs ICER (Class 4)
eFigure 12. One-Way Sensitivity Analysis Between Cost for Utility for Recurrent Cancer Post-LC vs ICER (Class 4)
eFigure 13. One-Way Sensitivity Analysis Between Cost for Utility for Post–Emergency Colectomy From LC vs ICER (Class 4)
eFigure 14. One-Way Sensitivity Analysis Between Monthly Mortality Rate vs ICER (Class 4)
eFigure 15. Probabilistic Sensitivity Analysis–ICER Scatterplot (Class 0)
eFigure 16. Probabilistic Sensitivity Analysis–Cost-effectiveness Acceptability Curve (Class 0)
eFigure 17. Probabilistic Sensitivity Analysis–ICER Scatterplot (Class 4)
eFigure 18. Probabilistic Sensitivity Analysis–Cost-effectiveness Acceptability Curve (Class 4)

This supplementary material has been provided by the authors to give readers additional information about their work.

© 2020 Jang SR et al. *JAMA Network Open*.

eFigure 1. SEER's CRC T1 Survival Curves With Linear Fit as Mortality Rates

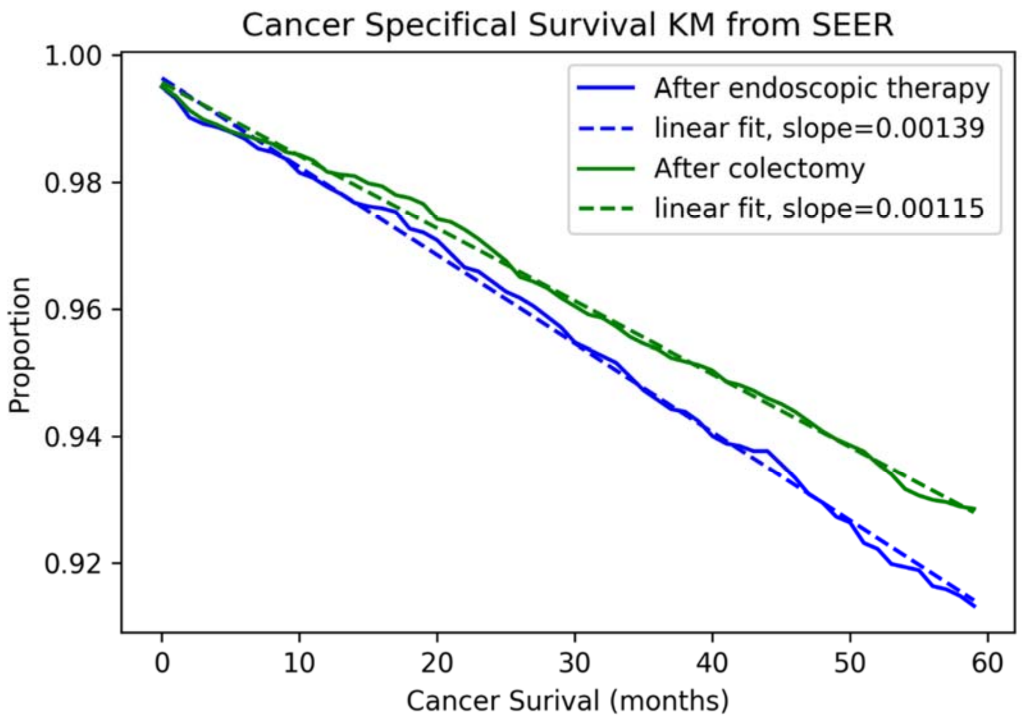

© 2020 Jang SR et al. *JAMA Network Open*.

eFigure 2. Calibrated Survival Curves Over Survival Curves From Schell et al.

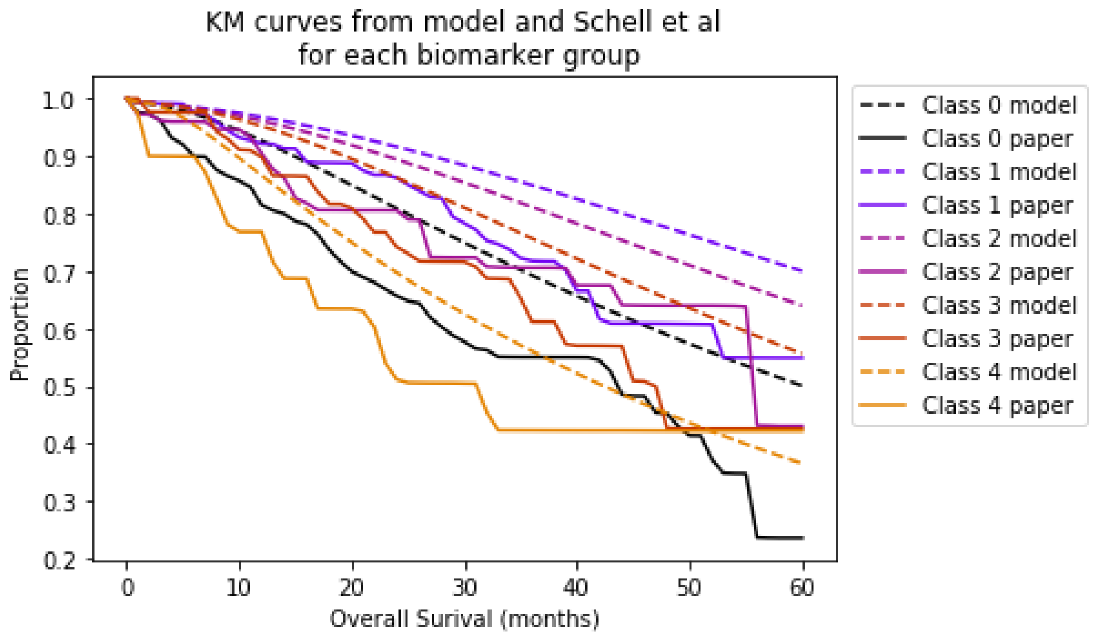

Dashed lines represent the survival curves calibrated by our research team.

© 2020 Jang SR et al. *JAMA Network Open*.

eFigure 3. One-Way Sensitivity Analysis Between Recurrent Cancer Treatment Cost Post-ET vs ICER (Class 0)

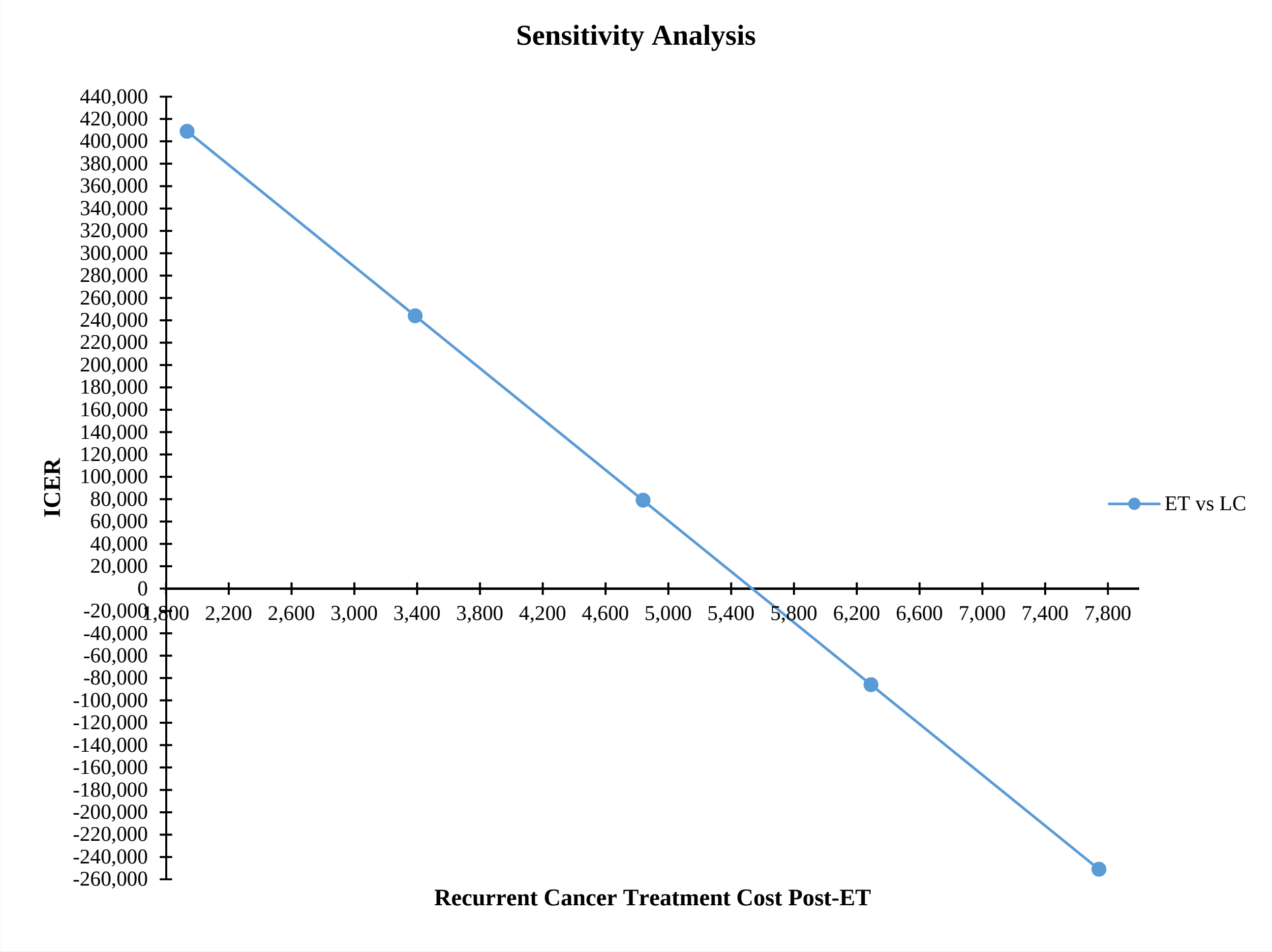

© 2020 Jang SR et al. *JAMA Network Open*.

eFigure 4. One-Way Sensitivity Analysis Between Cost for Recurrent Cancer Treatment Post-LC vs ICER (Class 0)

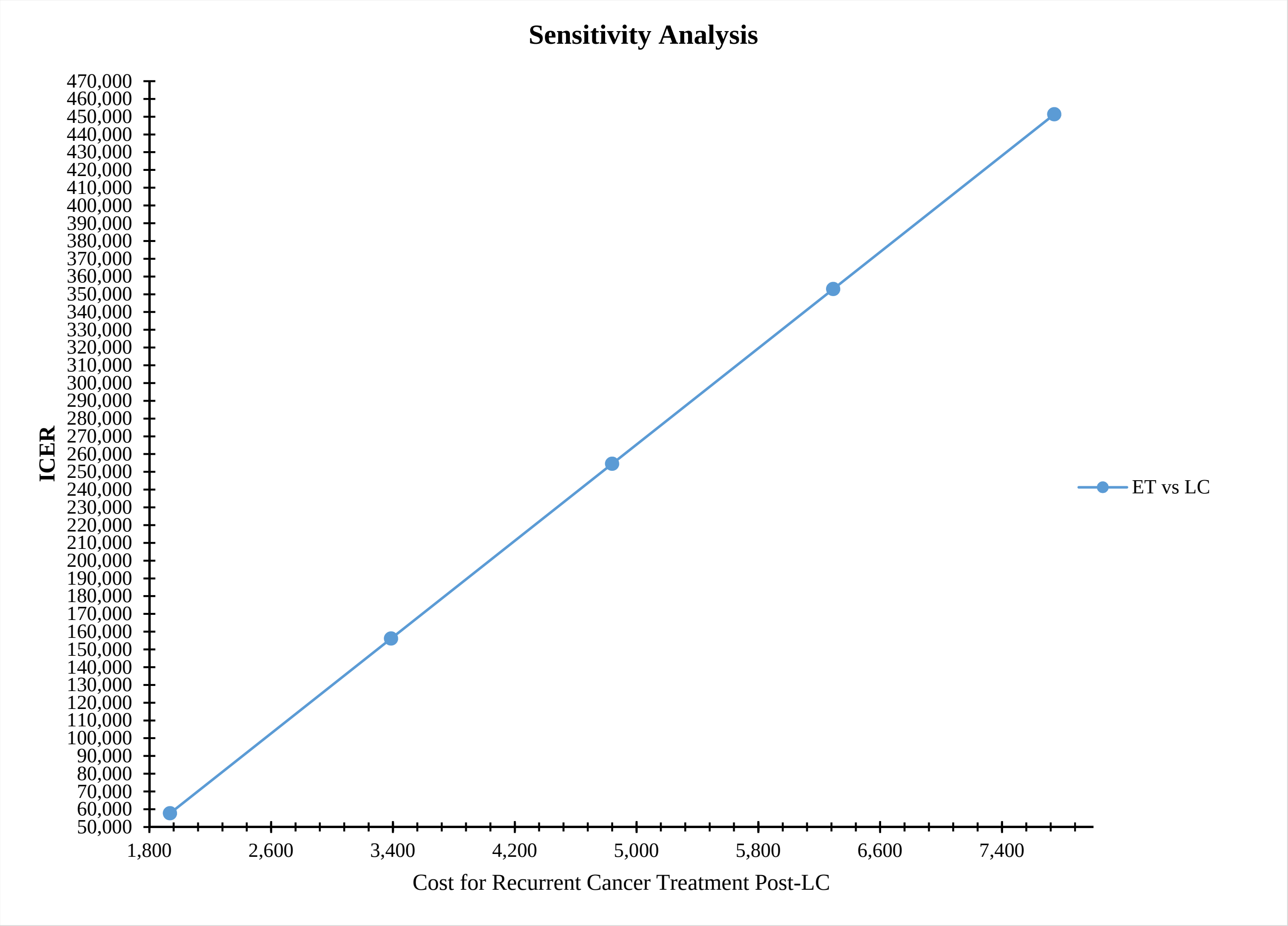

© 2020 Jang SR et al. *JAMA Network Open*.

eFigure 5. One-Way Sensitivity Analysis Between LC Treatment Cost vs ICER (Class 0)

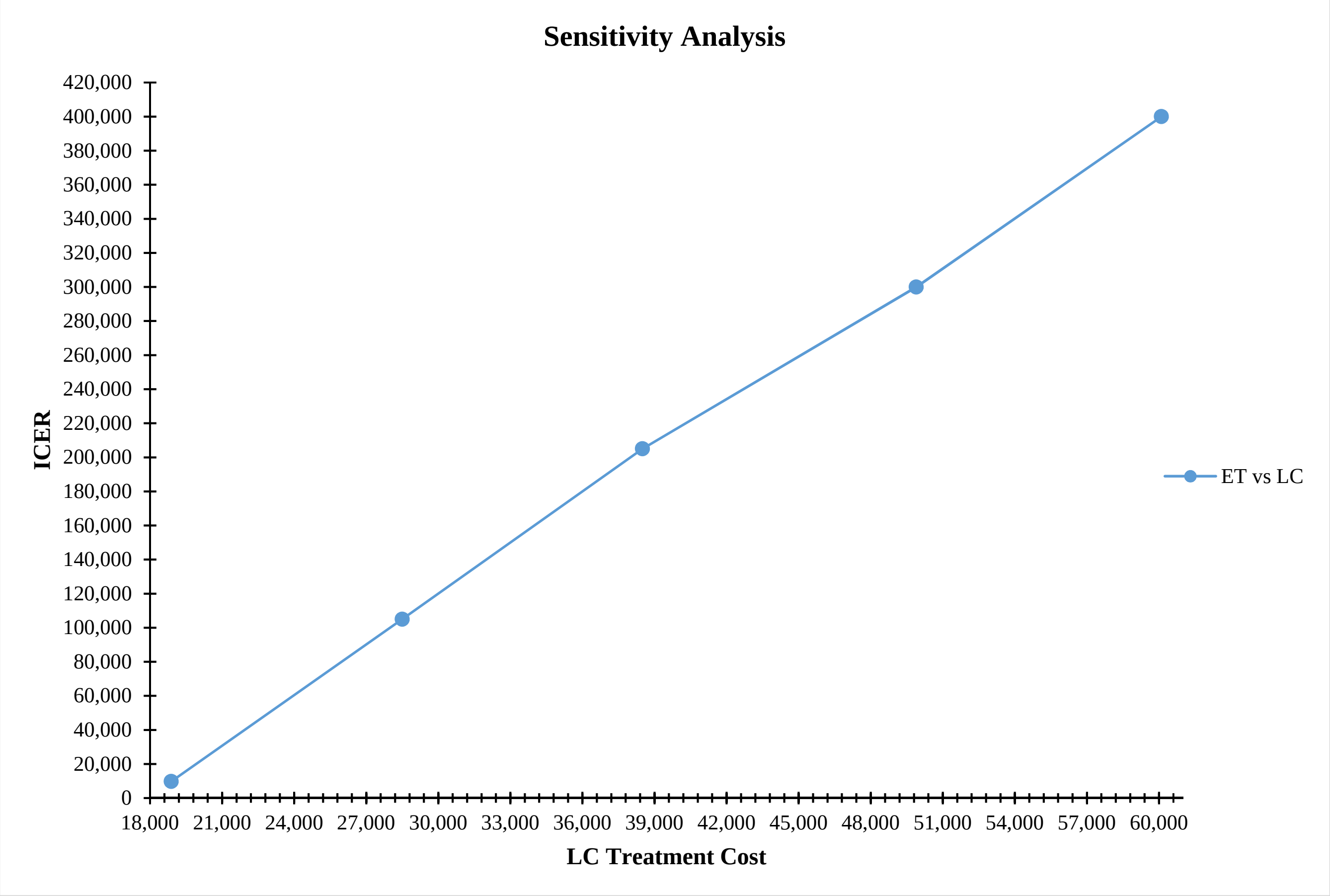

© 2020 Jang SR et al. *JAMA Network Open*.

eFigure 6. One-Way Sensitivity Analysis Between Monthly Recurrent Cancer Rate Post-LC vs ICER (Class 4)

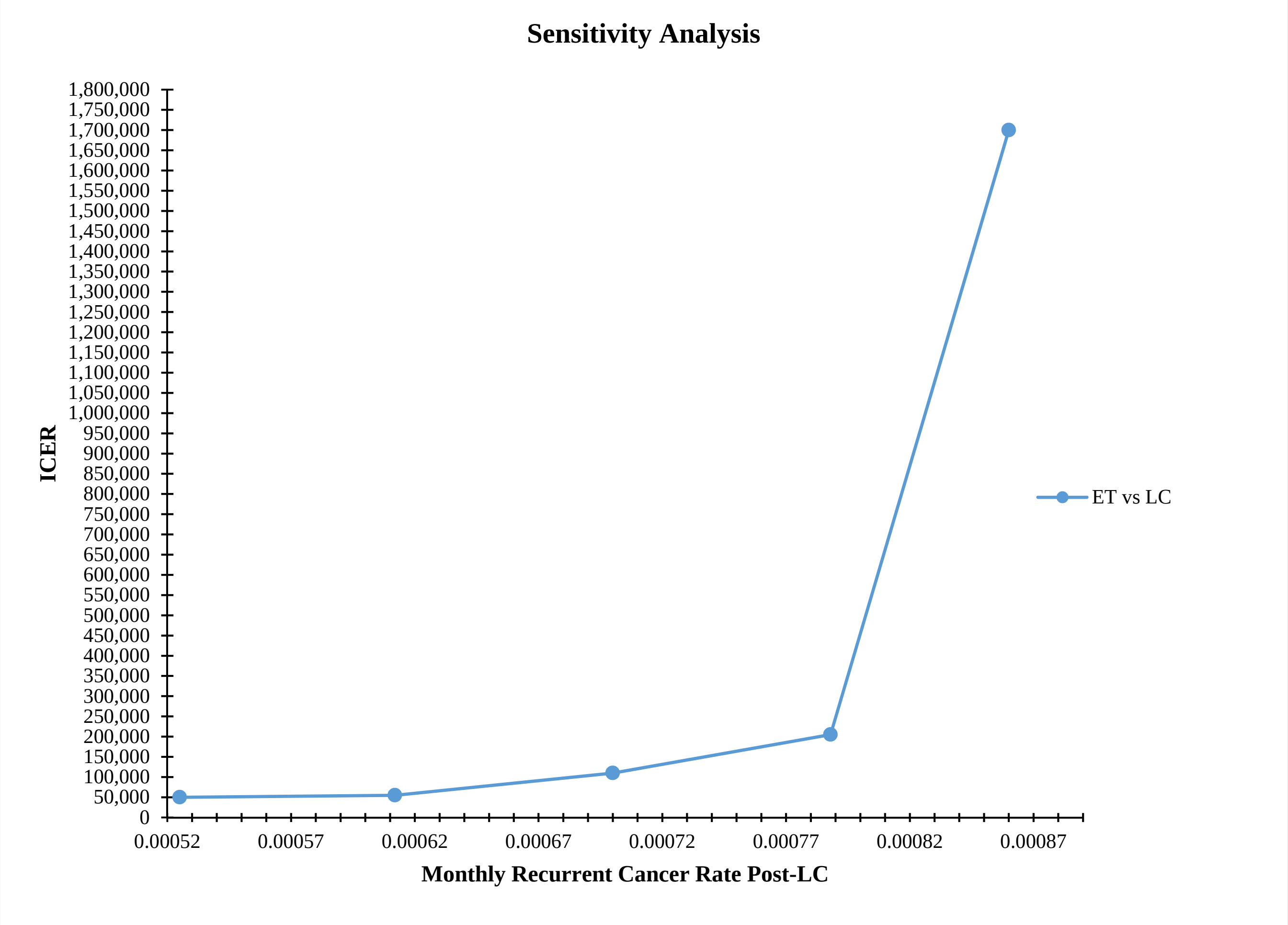

© 2020 Jang SR et al. *JAMA Network Open*.

eFigure 7. One-Way Sensitivity Analysis Between Monthly Recurrent Cancer Rate Post–Emergency Colectomy from LC vs ICER (Class 4)

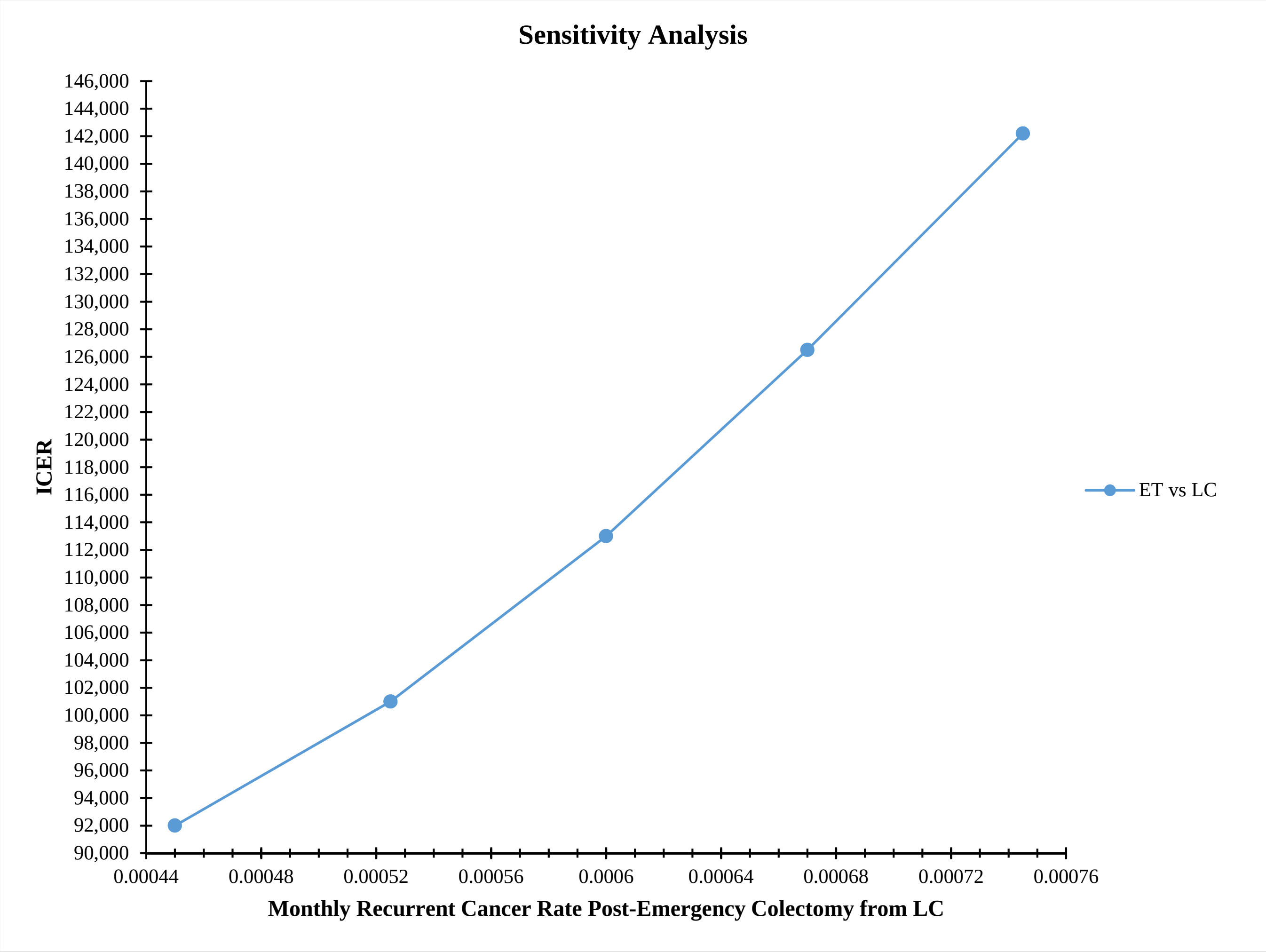

© 2020 Jang SR et al. *JAMA Network Open*.

eFigure 8. One-Way Sensitivity Analysis Between LC Cost vs ICER (Class 4)

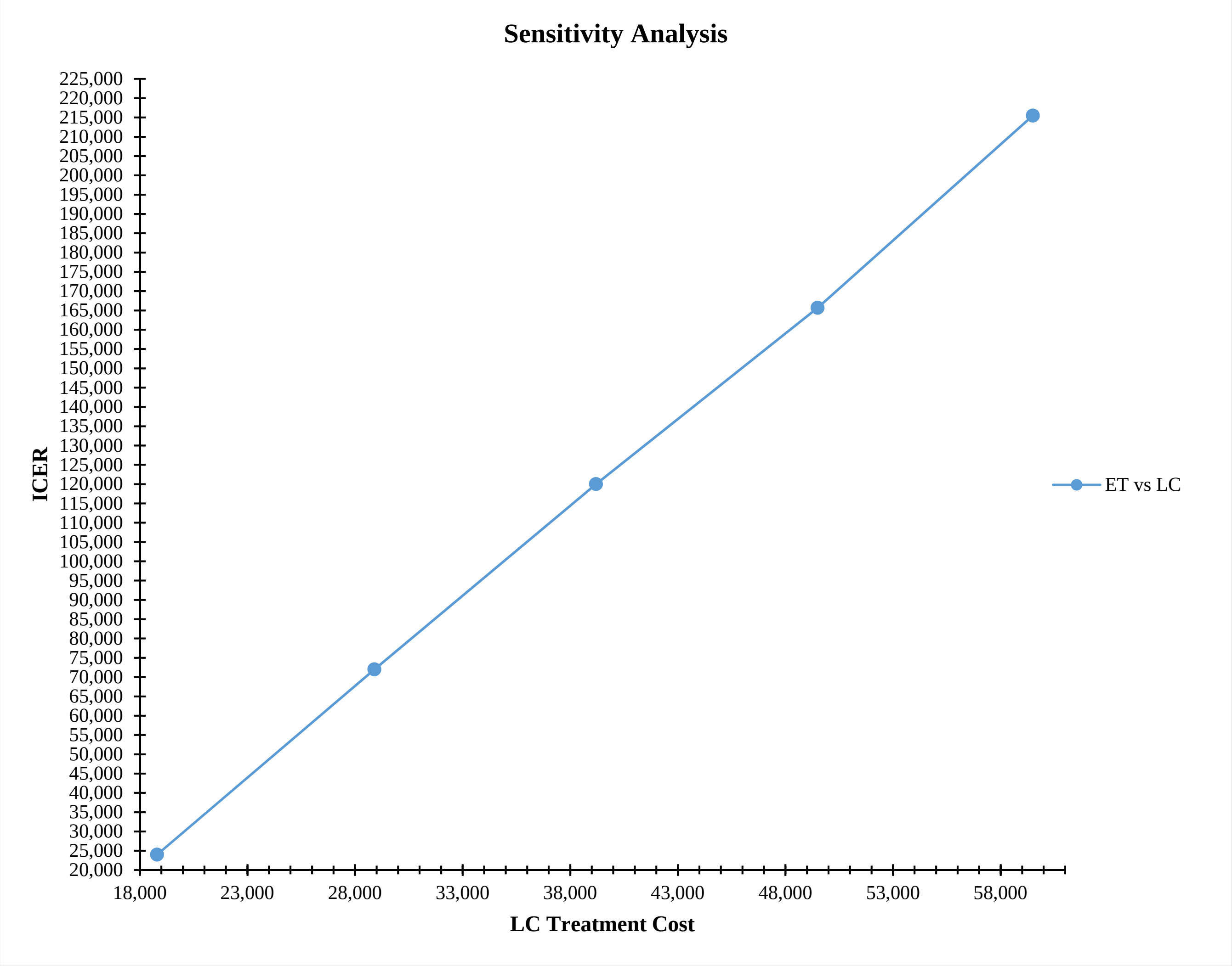

© 2020 Jang SR et al. *JAMA Network Open*.

eFigure 9. One-Way Sensitivity Analysis Between Cost for Recurrent Cancer Treatment Post-LC vs ICER (Class 4)

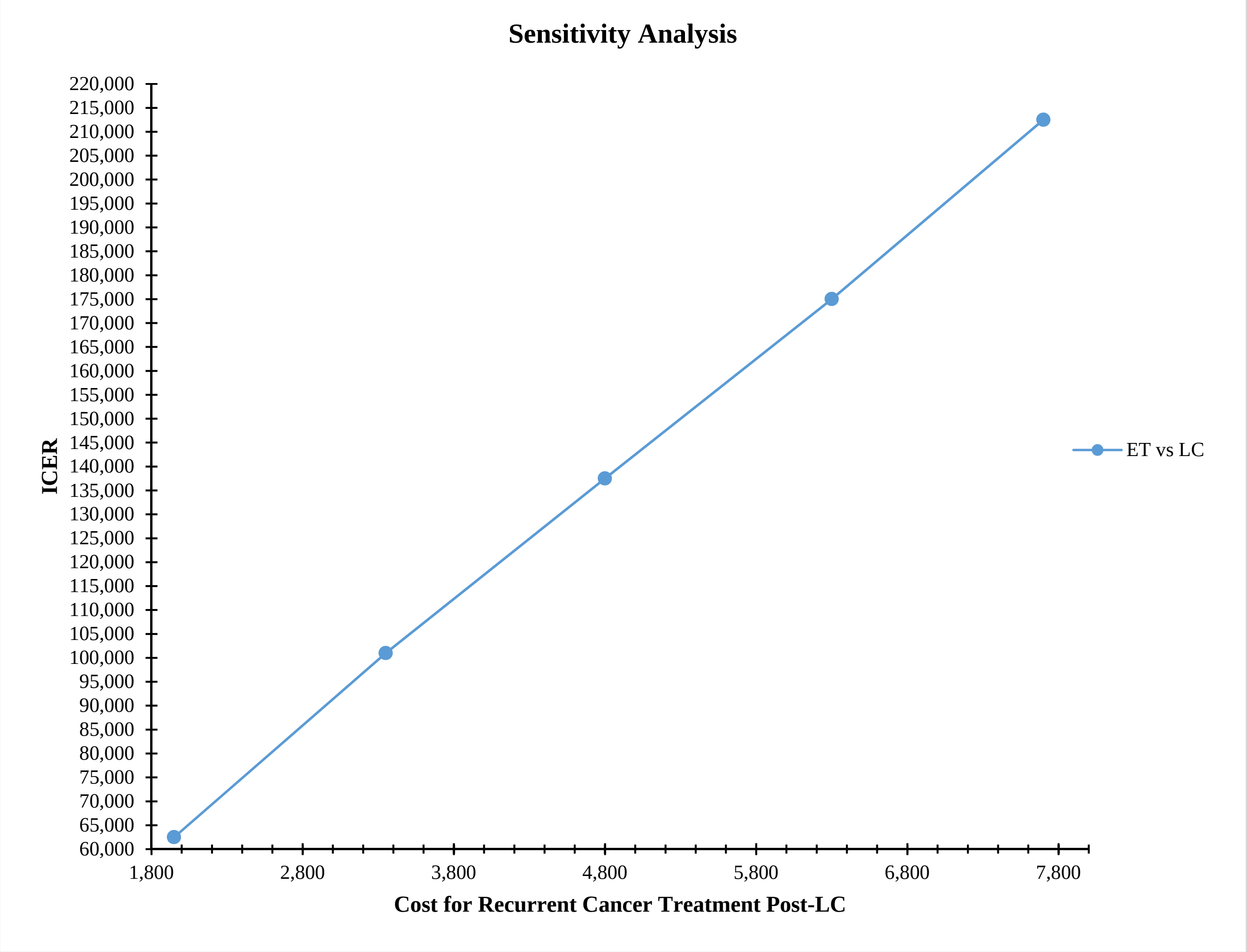

© 2020 Jang SR et al. *JAMA Network Open*.

eFigure 10. One-Way Sensitivity Analysis Between Cost for Recurrent Cancer Treatment Post-ET vs ICER (Class 4)

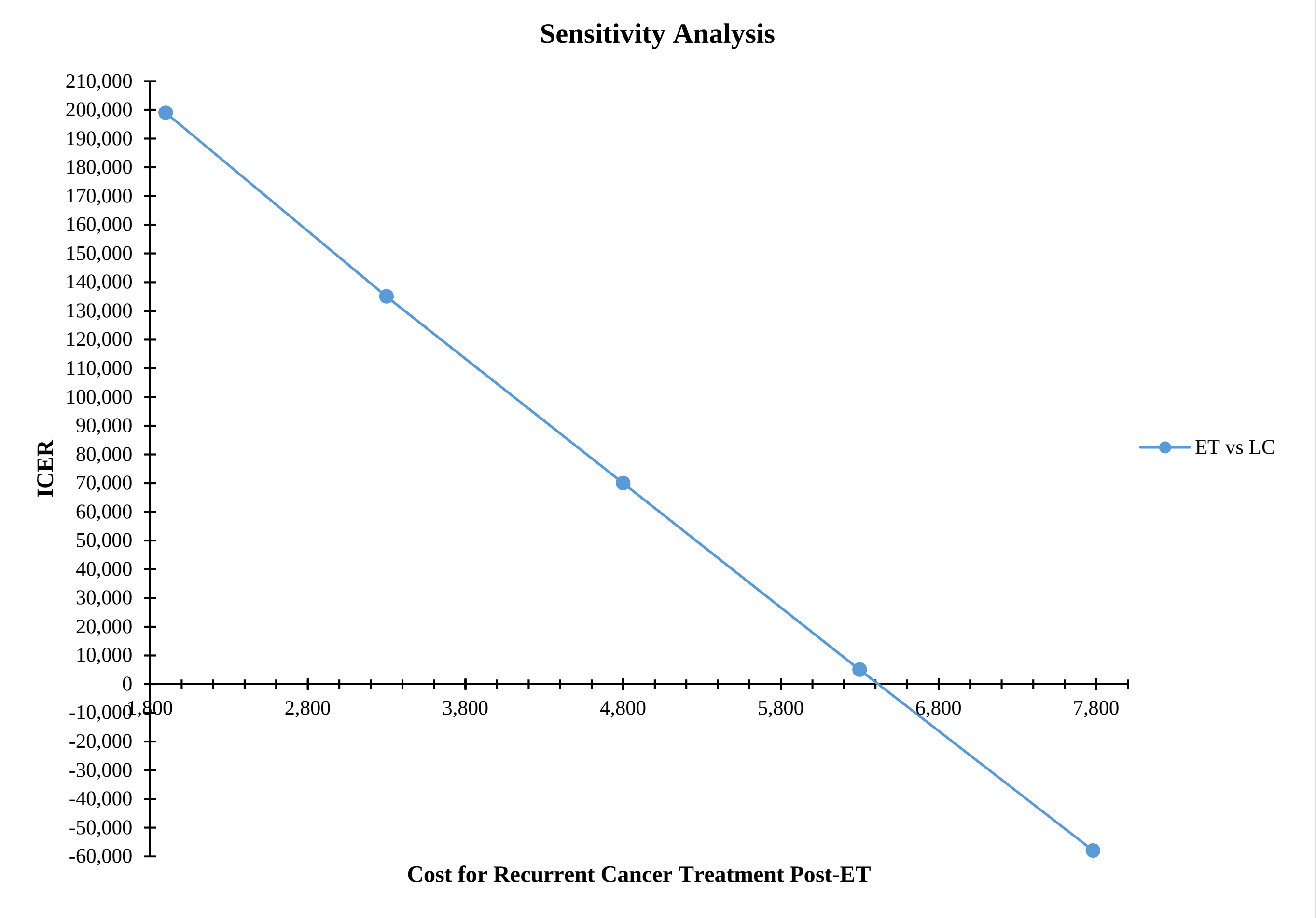

© 2020 Jang SR et al. *JAMA Network Open*.

eFigure 11. One-Way Sensitivity Analysis Between Utility for Recurrent Cancer Post-ET vs ICER (Class 4)

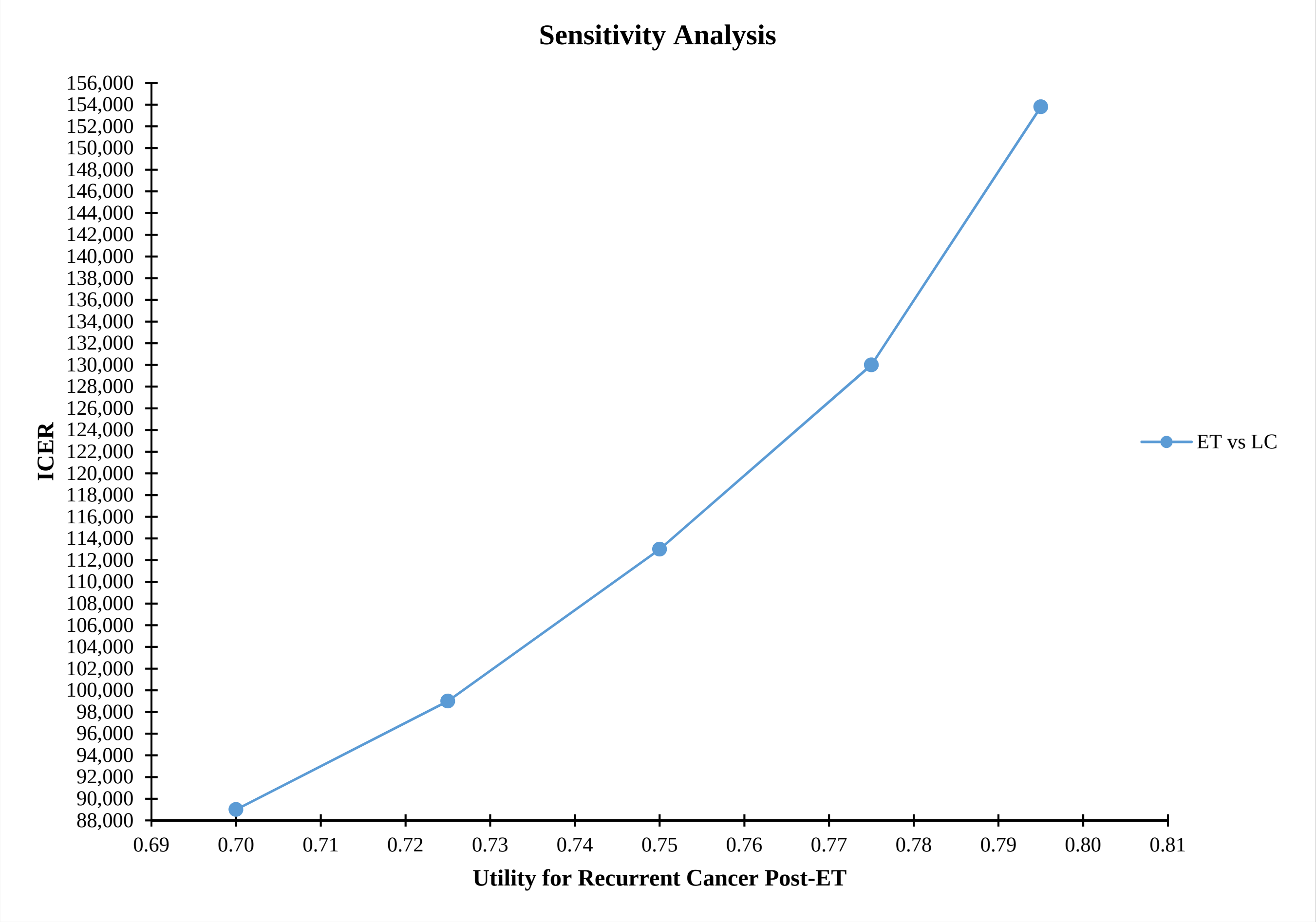

© 2020 Jang SR et al. *JAMA Network Open*.

eFigure 12. One-Way Sensitivity Analysis Between Utility for Recurrent Cancer Post-LC vs ICER (Class 4)

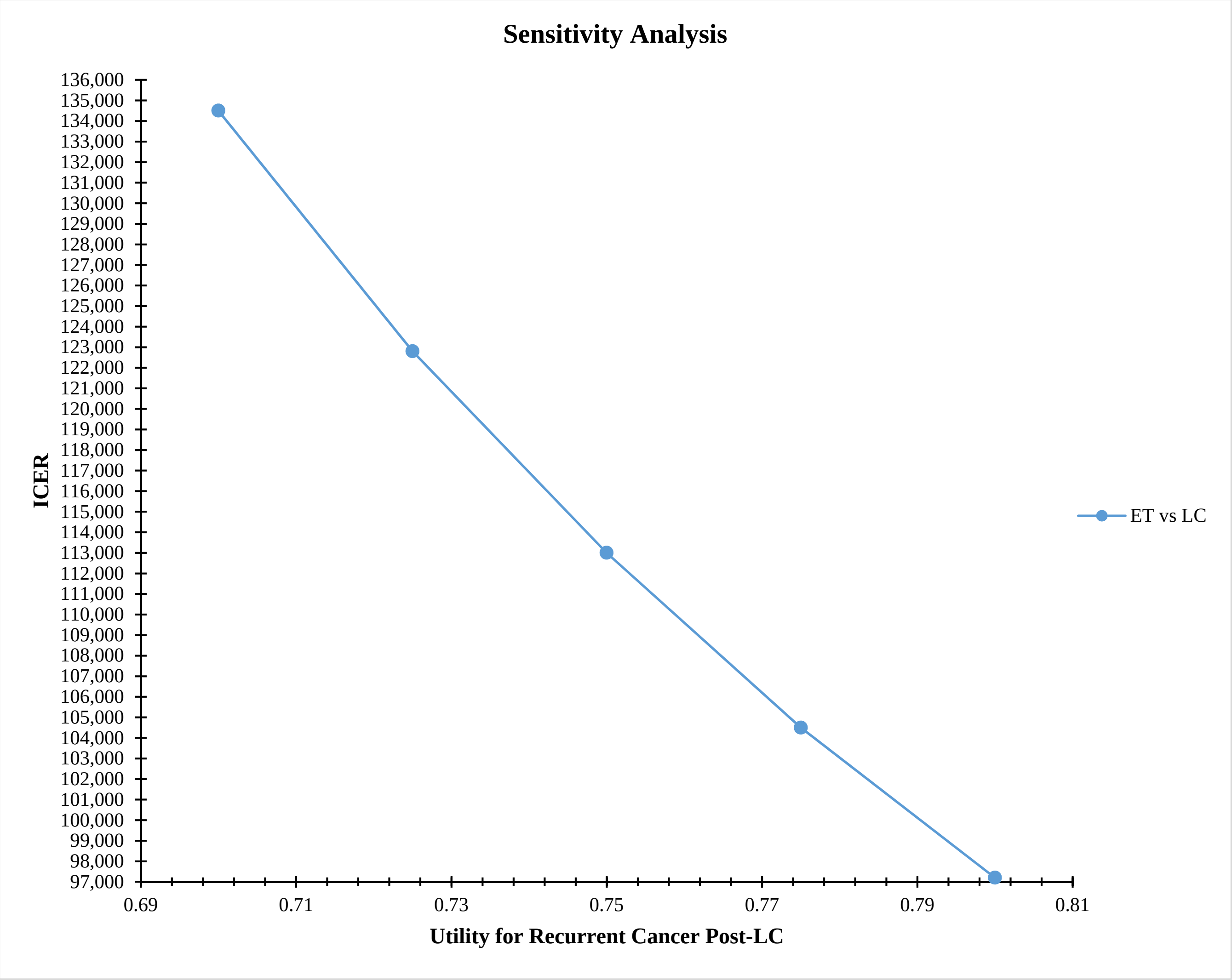

© 2020 Jang SR et al. *JAMA Network Open*.

eFigure 13. One-Way Sensitivity Analysis Between Utility for Post–Emergency Colectomy from LC vs ICER (Class 4)

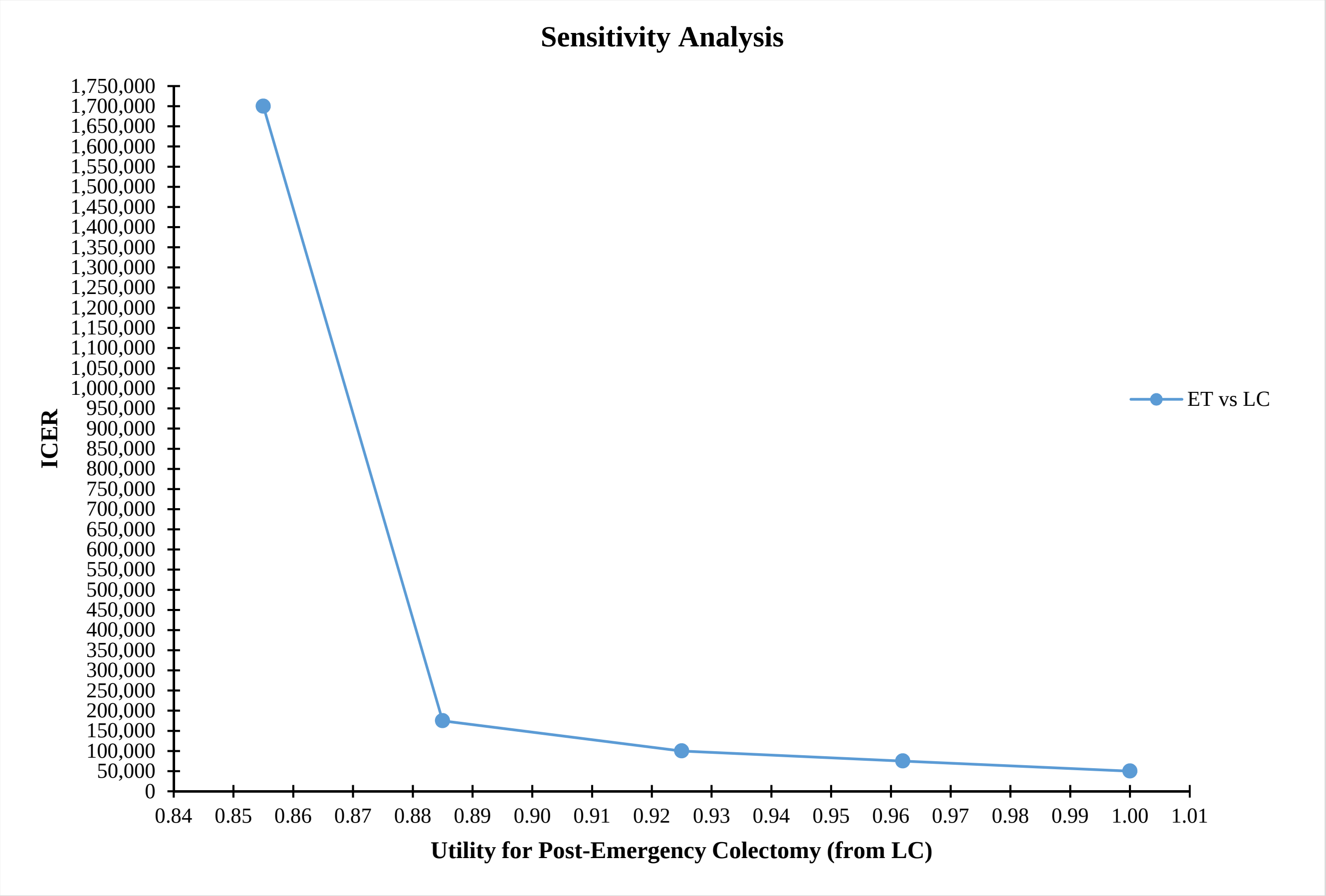

© 2020 Jang SR et al. *JAMA Network Open*.

eFigure 14. One-Way Sensitivity Analysis Between Monthly Mortality Rate vs ICER (Class 4)

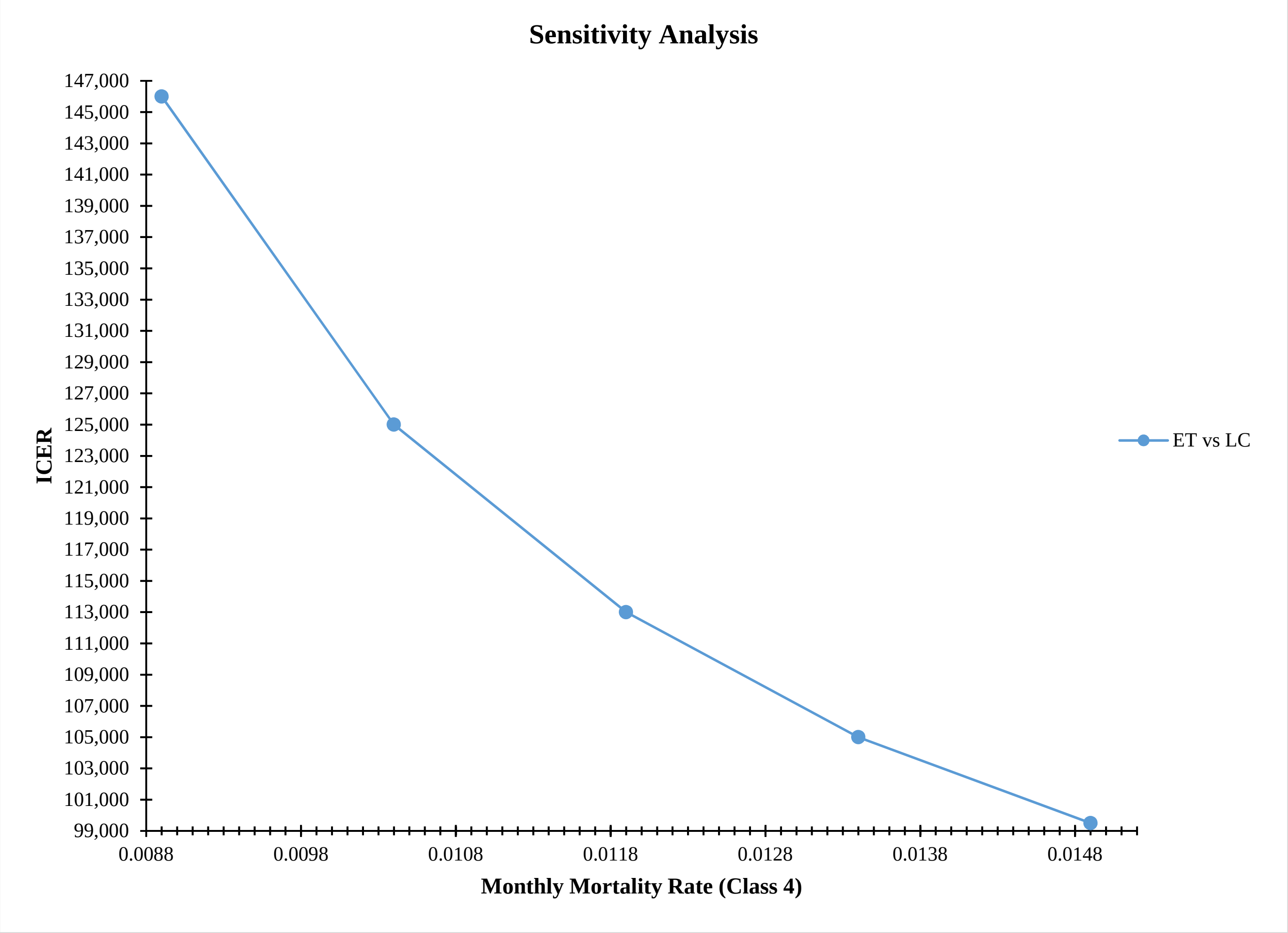

Abbreviations: LC, laparoscopic colectomy; ICER, incremental cost-effectiveness ratio; ET, endoscopic therapy

© 2020 Jang SR et al. *JAMA Network Open*.

eFigure 15. Probabilistic Sensitivity Analysis–ICER Scatter Plot (Class 0)

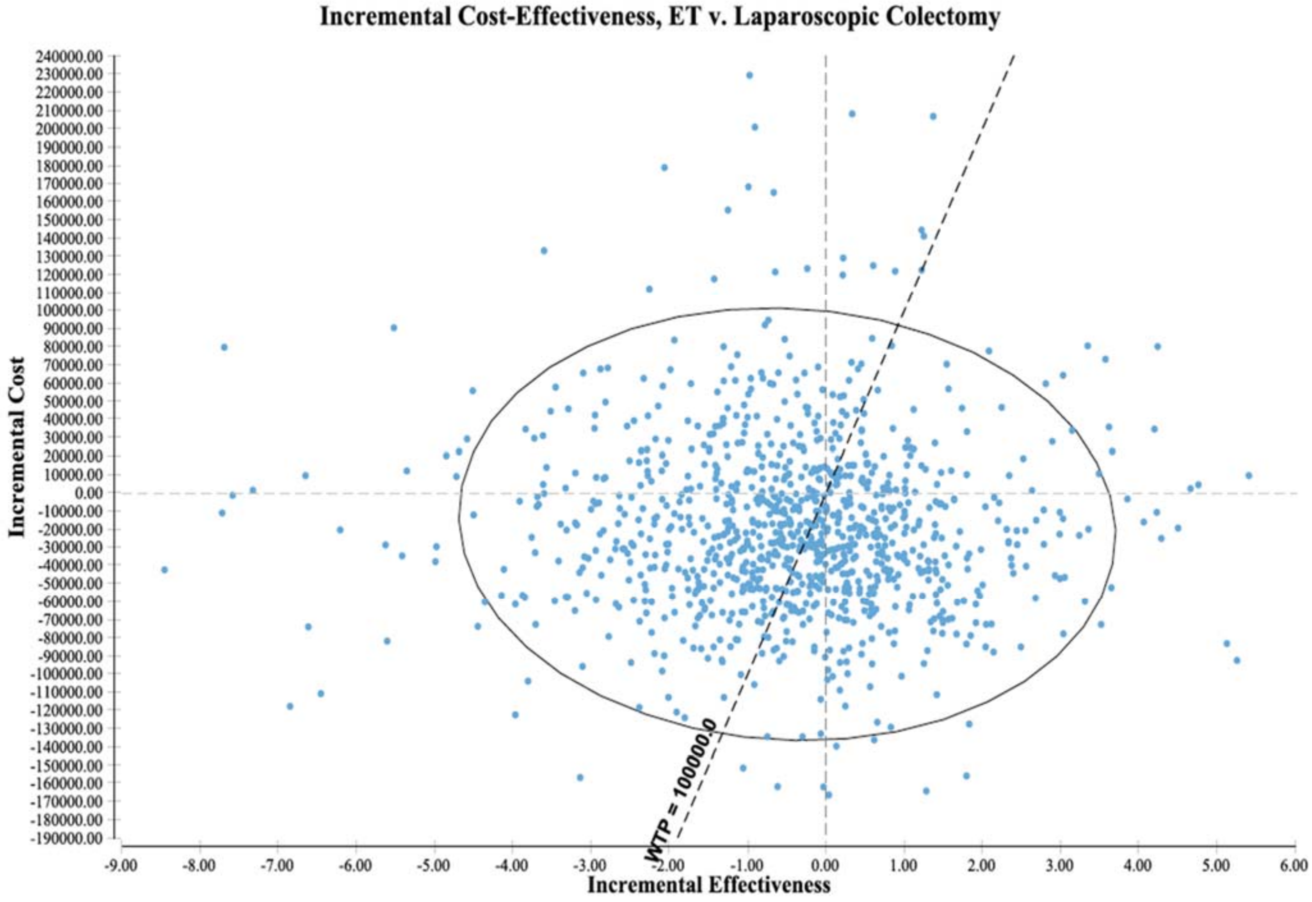

© 2020 Jang SR et al. *JAMA Network Open*.

eFigure 16. Probabilistic Sensitivity Analysis–Cost-effectiveness Acceptability Curve (Class 0)

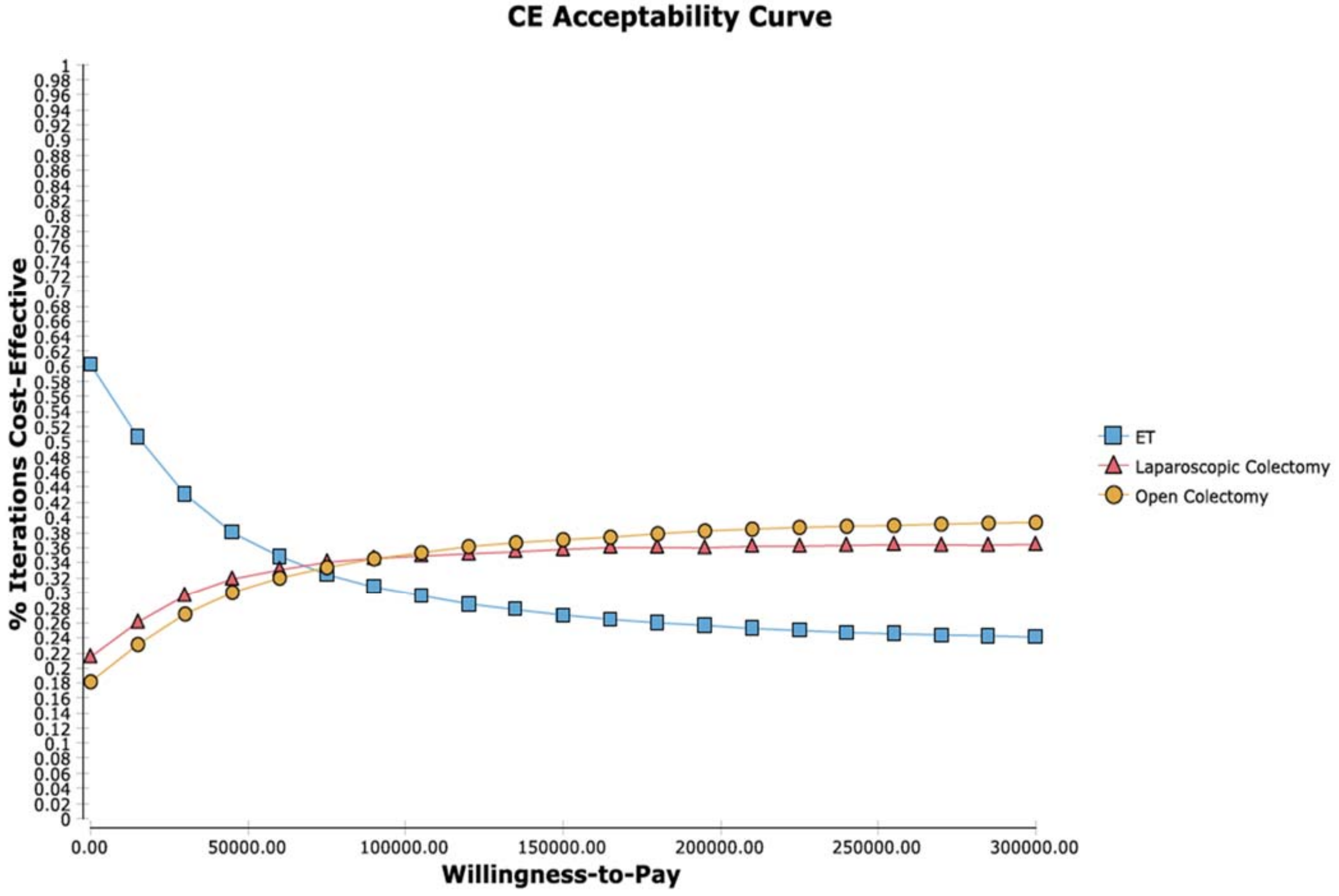

© 2020 Jang SR et al. *JAMA Network Open*.

eFigure 17. Probabilistic Sensitivity Analysis–ICER Scatterplot (Class 4)

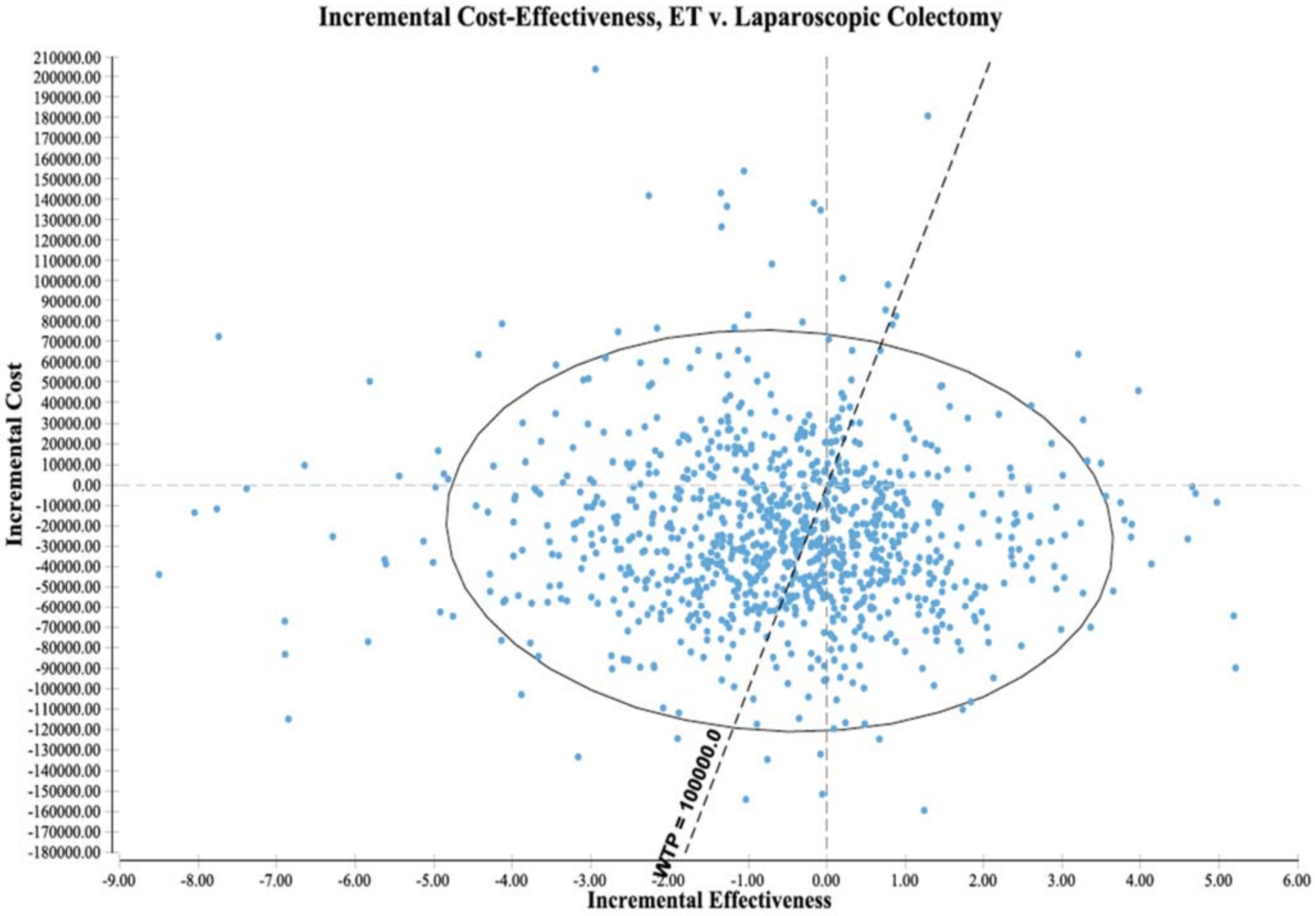

© 2020 Jang SR et al. *JAMA Network Open*.

eFigure 18. Probabilistic Sensitivity Analysis–Cost-effectiveness Acceptability Curve (Class 4)

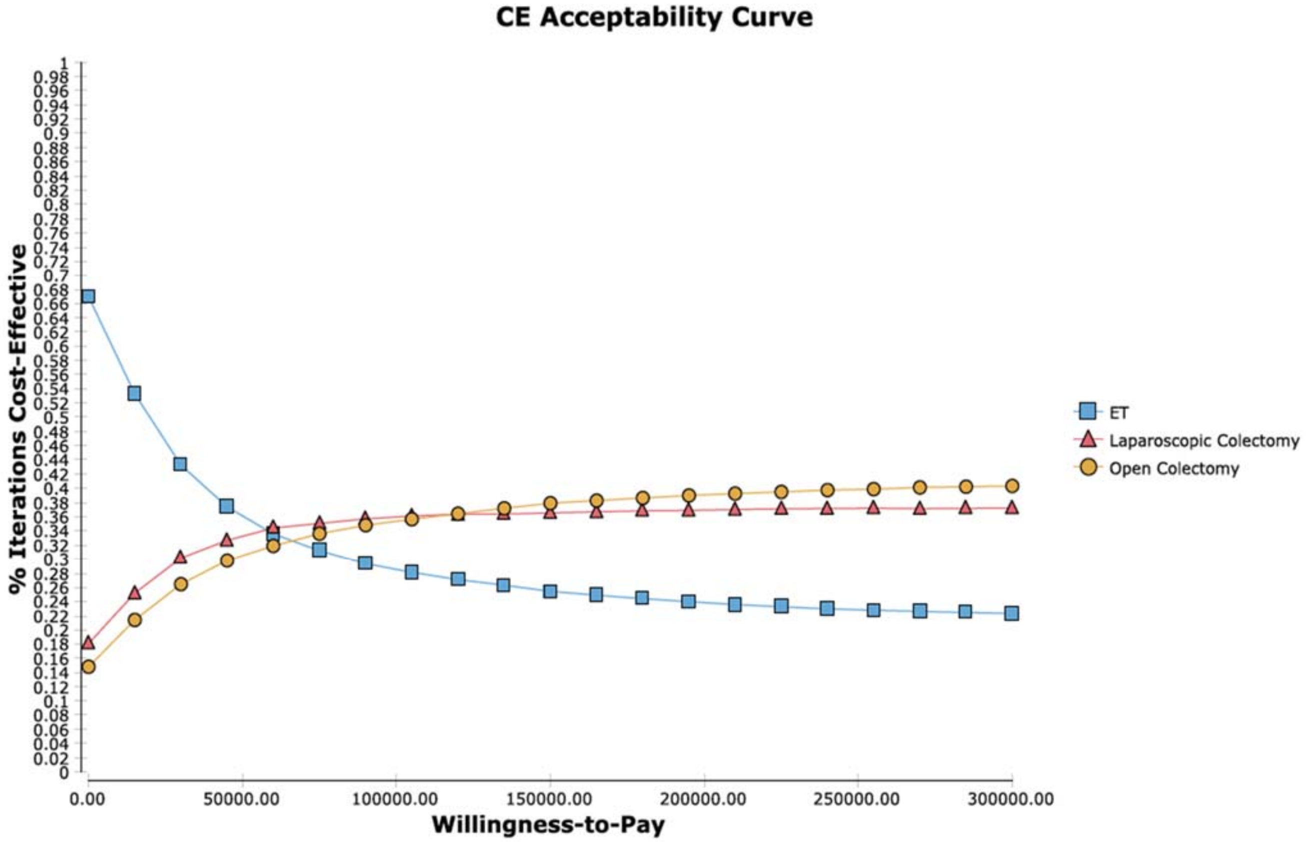

Abbreviations: ET, endoscopic therapy; LC, laparoscopic colectomy; ICER, incremental cost-effectiveness ratio

© 2020 Jang SR et al. *JAMA Network Open*.
